# Integrated Clinical, Molecular, and Machine Learning Assessment of Familial Hypercholesterolemia

**DOI:** 10.3390/life16040633

**Published:** 2026-04-09

**Authors:** Mustafa Tarık Alay, Atakan Deniz, Hanife Saat, Haktan Bağış Erdem

**Affiliations:** Department of Medical Genetics, Ankara Etlik City Hospital, 06170 Ankara, Türkiye; atakandenizmd@gmail.com (A.D.); hanifesaat@gmail.com (H.S.); haktanbagis@gmail.com (H.B.E.)

**Keywords:** Simon Broome, machine learning, familial hypercholesterolemia, familial hypertriglyceridemia

## Abstract

**Background**: In clinical practice, LDL-dominant familial hypercholesterolemia (FH) may overlap phenotypically with triglyceride-dominant or mixed familial dyslipidemia. Rule-based diagnostic approaches like the Dutch Lipid Clinic Network (DLCN) and Simon Broome (SB) criteria are frequently used in countries with limited genetic testing, but their concordance with molecular confirmation is inconsistent. In a large Turkish tertiary-care cohort, we studied phenotype-related discordance between clinical criteria and molecular data and tested whether machine learning (ML) models could improve the prediction of reportable pathogenic/likely pathogenic variant positivity among patients with a clinical FH phenotype. **Methods**: Patients referred for suspected familial hyperlipidemia underwent targeted next-generation sequencing with a 9-gene panel. For the ML analysis, we focused on FH cases with a definitive molecular status (pathogenic/likely pathogenic vs. no reportable variant; variants of uncertain significance were excluded) and applied an 80/20 stratified split (*n* = 200; 82 molecular-positive cases). Elastic-net logistic regression, random forest, and XGBoost models trained on routinely available clinical variables were compared with dichotomized SB and DLCN classifications. **Results**: SB positivity was significantly more frequent in triglyceride-dominant phenotypes than in FH (68.4% vs. 52.3%, *p* = 0.041), despite the substantially lower molecular positivity (14.0% vs. 36.9%, *p* = 0.002), indicating FH-like false-positive clinical classification in mixed dyslipidemia. In the FH test set, the ML models showed higher discrimination for reportable pathogenic/likely pathogenic variant positivity than dichotomized rule-based criteria (AUC: XGBoost 0.808; random forest 0.769; elastic-net 0.747 vs. SB 0.639; and DLCN 0.598). Thirteen novel variants absent from gnomAD were identified, predominantly in *LDLR*. **Conclusions**: In this real-world Turkish cohort, within clinically defined FH cases, ML models performed better at predicting LP/P variant positivity than dichotomized DLCN and Simon Broome criteria. ML-based risk stratification may support prioritization for genetic testing; however, external validation is warranted.

## 1. Introduction

Familial hyperlipidemias represent a heterogeneous group of inherited metabolic disorders characterized by elevated levels of lipids, particularly cholesterol and triglycerides, in the bloodstream. These genetic conditions often lead to premature development of cardiovascular diseases, including coronary artery disease and atherosclerosis [1]. It is predominantly inherited in an autosomal dominant manner, affecting approximately 1 in 200 to 300 individuals in the general population [2,3]. The implementation of screening methods across different countries has not solved the problem of undetected cases [4]. This underdiagnosis is particularly concerning given that early detection and intervention can significantly mitigate the long-term sequelae of the disease [5]. The pathogenic variants responsible for the disease are primarily located in three main genes: low-density lipoprotein receptor (*LDLR*), apolipoprotein B (*APOB*), and proprotein convertase subtilisin/kexin type 9 (*PCSK9*). These genes account for approximately 85–90%, 5–10%, and 1–3% of all variants causing FH, respectively. The penetrance of pathogenic and likely pathogenic variants in these genes ranges from 25% to 90% [6,7,8,9].

Familial hypercholesterolemia is diagnosed primarily on clinical grounds using validated criteria. Genetic testing, when available, provides etiologic confirmation and facilitates cascade screening, but it is not required for diagnosis, and failure to detect a pathogenic/likely pathogenic variant does not exclude FH. Since genetic testing is often not feasible, the diagnosis of FH is usually based on clinical findings. The Dutch Lipid Clinic Network (DLCN) and Simon Broome criteria, commonly used in the diagnosis of familial hypercholesterolemia (FH), can be applied to both children and adults. These criteria evaluate parameters such as physical examination findings, serum cholesterol levels, genetic testing, and family history [10,11]. On the other hand, triglyceride-dominant or mixed dyslipidemia, including familial combined hyperlipidemia and some familial hypertriglyceridemia presentations, is an important differential diagnosis in patients referred with suspected familial dyslipidemia because these phenotypes may overlap clinically with FH-like presentations. This distinction is relevant when rule-based systems are applied, as triglyceride-rich or mixed dyslipidemia patterns may increase clinical suspicion of FH without corresponding molecular confirmation [12,13].

Traditional classification schemes for dyslipidemia have become outdated, giving way to approaches centered on the primary lipid disturbance identified through routine lipid panels, thus offering a more practical starting point for diagnosis and management [14]. The advent of next-generation sequencing (NGS) has revolutionized human genetics by enabling the rapid and cost-effective analysis of multiple genes simultaneously. This advancement has opened unprecedented opportunities for accurate diagnosis, risk stratification, and personalized treatment strategies [15,16,17,18]. NGS technologies have become powerful tools for identifying causative genetic variants and dissecting the genetic architecture of complex diseases such as hyperlipidemia, allowing for the detection of both rare and common variants that contribute to disease susceptibility [19].

In the management of atherosclerotic diseases, LDL-cholesterol-lowering therapies (statins, ezetimibe, bempedoic acid, colesevelam, and PCSK9 inhibitors) can be used alone or in combination, while lifestyle changes and other ASCVD risk factors can additionally be targeted. Therefore, accurate and early diagnosis is a critical step for patient prognosis. However, genetic confirmation can be achieved in only 40–70% of patients who are clinically suspected of having FH based on established diagnostic criteria [20]. Although current diagnostic algorithms provide an important guide, the development of new methods to improve diagnostic accuracy is inevitable. In the era of artificial intelligence, the use of machine learning algorithms integrated with traditional criteria has the potential to not only improve diagnostic performance but also provide additional benefits in terms of patient management. The primary objective of this study was to examine discordance between clinical rule-based criteria and molecular findings in patients with suspected familial dyslipidemia and, as a prespecified secondary analysis, to assess whether machine learning models improve the prediction of reportable pathogenic/likely pathogenic variant positivity within the clinically defined FH subset.

## 2. Materials and Methods

### 2.1. Patient Selection

A total of 126,097 patients were evaluated for hypercholesterolemia, and patients with suspected familial hypercholesterolemia following a multidisciplinary evaluation (pediatric or adult cardiologist, pediatric geneticist or clinical geneticist, and internal medicine specialists) were referred to Ankara Etlik City Hospital Medical Genetics Department. Ankara Etlik City Hospital is one of the largest tertiary care centers in Turkey. Located in Ankara, it serves a broad referral population, predominantly from Central Anatolia as well as other regions of the country. The hospital has a very high daily patient volume, with approximately 30,000–40,000 outpatient visits per day across all specialties. Patients recruited between 2022 and 2025 were retrospectively/prospectively evaluated, and the age range was restricted to 0–75 years. Written informed consent was obtained from all participants in accordance with the Declaration of Helsinki.

Because this study was conducted at a single tertiary referral center in Ankara, we additionally evaluated geographic origin data recorded for the patients to contextualize potential referral clustering within the cohort [21,22]. This analysis was exploratory and was not intended to estimate regional prevalence; rather, it was performed to aid in the interpretation of cohort representativeness and generalizability. Geographic origin was summarized descriptively according to the major geographic regions of Türkiye using routinely recorded birthplace/province data where available. Participants of international origin were excluded from the map-based summary to preserve domestic geographic comparability.

### 2.2. Patient Evaluation

#### Diagnostic Criteria

Patients referred to our genetic diagnosis and treatment center were evaluated if they had both a family history of hyperlipidemia and elevated low-density lipoprotein (LDL) cholesterol suggestive of familial hypercholesterolemia. Afterwards, all referred patients were initially screened for familial hypercholesterolemia (FH) using the MEDPED diagnostic thresholds, which incorporate age- and family history-based cholesterol cut-offs (Supplementary Table S1). Individuals who fulfilled the MEDPED thresholds and were considered to be at risk for FH subsequently underwent further evaluation according to the Dutch Lipid Clinic Network (DLCN) and Simon Broome criteria scoring system (Supplementary Tables S2 and S3, respectively). The diagnostic criteria were applied as previously described [2,23,24]

The DLCN and Simon Broome criteria are the two most commonly used systems for diagnosing FH, incorporating different age groups and different clinical weightings. The rarity of physical examination findings (e.g., tendon xanthomas) in pediatric cases and the variability of LDL thresholds according to age may affect the performance of these diagnostic systems. Therefore, simultaneously reporting the scores of both systems allows for (i) comparing these two main approaches used by clinicians in real life within the same cohort, (ii) revealing possible systematic deviations across the pediatric–adult spectrum, and (iii) highlighting discordance between the two diagnostic systems.

### 2.3. FH Diagnosis

Clinical FH classification was assessed using both the DLCN and Simon Broome criteria. For descriptive purposes, DLCN categories were recorded as unlikely, possible (DLCN score 3–5), probable (DLCN score 6–8), or definite (DLCN score > 8), and binary classifications were generated for comparative analyses using the predefined study cut-offs [2,5,24].

The DLCN [24] and Simon Broome criteria [8,24,25], commonly used in the diagnosis of familial hypercholesterolemia (FH), can be applied to both children and adults. Age-specific LDL-cholesterol and total cholesterol thresholds are defined in these systems as follows: the DLCN uses lower cut-offs for children and higher cut-offs for adults, while the Simon Broome criteria use the thresholds of total cholesterol > 6.7 mmol/L or LDL > 4.0 mmol/L for children < 16 years of age and total cholesterol > 7.5 mmol/L or LDL > 4.9 mmol/L for adults. These parameters, along with clinical findings and family history, enable patients to be classified as having “definite” or “probable” FH.

### 2.4. Molecular Diagnosis

**Figure 1:**
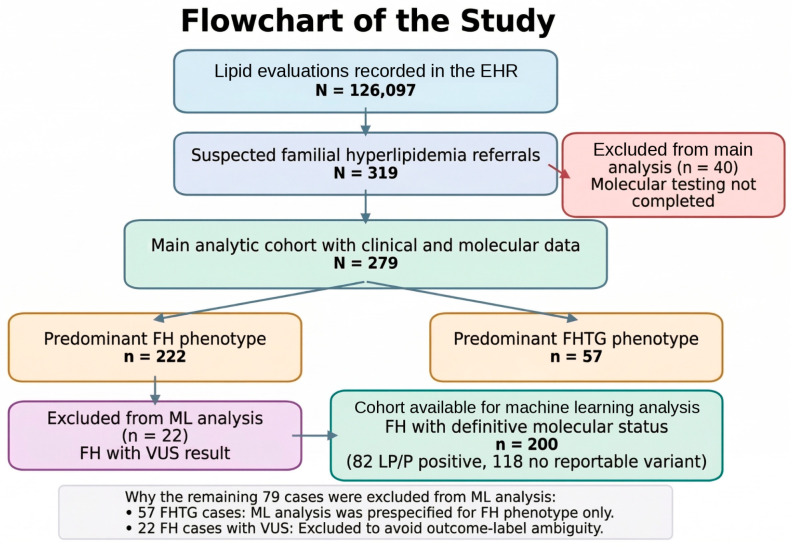
Flowchart of the study. A total of 319 patients referred to the Medical Genetics Department for suspected familial hyperlipidemia were initially assessed. Forty patients were excluded because molecular testing had not been completed at the time of data lock, leaving 279 individuals with available clinical and molecular data for the main analyses. Patients were then categorized according to the predominant lipid phenotype as familial hypercholesterolemia (FH; n = 222) or familial hypertriglyceridemia (FHTG; n = 57). Clinical classification was evaluated using the MEDPED, Simon Broome (SB), and Dutch Lipid Clinic Network (DLCN) criteria. For binary analyses, SB positivity was defined as possible/definite FH, and DLCN positivity was defined as definite/probable/possible FH. Machine learning analyses were restricted to FH cases with definitive molecular status; after exclusion of variants of uncertain significance (VUS; n = 22), 200 patients remained (82 molecular-positive and 118 molecular-negative) for the 80/20 stratified train/test split. FH, familial hypercholesterolemia; FHTG, familial hypertriglyceridemia; SB, Simon Broome; DLCN, Dutch Lipid Clinic Network; VUS, variant of uncertain significance; NGS, next-generation sequencing.

The hyperlipidemia gene panel covered 9 genes (*APOA5*, *APOB*, *CFH*, *GLA*, *GNAS*, *LDLR*, *LPL*, *MC4R*, and *PCSK9*), which were chosen to represent genes implicated in familial hypercholesterolemia (n = 3), familial hypertriglyceridemia (n = 2), and selected monogenic disorders associated with secondary or syndromic hyperlipidemia. A hyperlipidemia panel was requested for patients who were deemed appropriate for the test by at least two genetic physicians. The flowchart of the study is shown in Figure 1. Baseline demographic characteristics (age and sex distribution) were recorded. Clinical lipid measurements represented the values available in the medical record at referral/sampling and were not uniform pretreatment values, because untreated baseline lipid data were not consistently available for all participants. Lipid-lowering treatment status at the time of sampling was therefore recorded and retained as a clinical variable in subsequent analyses.

### 2.5. Molecular Evaluation

#### 2.5.1. DNA Isolation

Genomic DNA was isolated from EDTA-anticoagulated whole-blood samples using the Zeesan automated isolation robot. The procedure relies on selective binding of DNA to magnetic beads, followed by sequential washing steps to remove contaminants and elution for recovery. DNA yield and purity were assessed with a Nanodrop spectrophotometer by recording the 260/280 and 260/230 absorbance ratios, as well as the microconcentration values. Only DNA samples fulfilling the quality thresholds were selected for library preparation, with a total target size of ~120 kb. The minimum library concentration required was 2 ng/µL.

#### 2.5.2. Library Preparation

Sequencing libraries were constructed on the Seq Genomize v8.2.3 platform (Roche) using the KAPA HyperCap Custom Kit. The workflow comprised DNA fragmentation, end-repair, A-tailing, adaptor ligation, PCR amplification, and target enrichment of selected genomic regions.

#### 2.5.3. Next-Generation Sequencing

The prepared libraries were sequenced on the Illumina NextSeq 2000 system, which employs bridge amplification of DNA fragments immobilized on the flow cell surface and synthesis by sequencing (SBS) chemistry using reversible terminator nucleotides with fluorescence-based detection. Sequencing was performed using paired-end reads (2 × 150 bp). The average on-target depth was >100×, with >95% of bases covering at least 30×.

#### 2.5.4. Bioinformatic Analysis

Raw reads were processed using the Seq Genomize cloud-based pipeline (https://seq.genomize.com.tr/panel/#/, accessed on 15 November 2025) and aligned to the GRCh38 (hg38) human reference genome. Variants were retained for interpretation only if they achieved a minimum per-allele coverage of 30× depth. Variants with an allele frequency > 0.005 in population databases (gnomAD, 1000 Genomes, ExAC, ESP) were excluded from the evaluation. Variant databases such as ClinGen, ClinVar, Human Genetic Mutation Databases (HGMD), and LOVD, along with the literature, were utilized for the evaluation of variant pathogenicity. The ClinGen Familial Hypercholesterolemia Expert Panel Specifications to the American College of Medical Genetics and Genomics (ACMG)/Association for Molecular Pathology (AMP) Variant Classification Guidelines Version 1.2 and ACMG 2015 criteria were used for the classification of hyperlipidemia gene panel variants. Variant calling was performed with BWA-MEM (v0.7.17) and GATK (v4.2.6.1). Annotation was conducted using ANNOVAR (v2022-07) and dbNSFP (v4.3a). Variants were filtered based on a minimum base quality (Q ≥ 30) and mapping quality (MQ ≥ 40). Pathogenicity prediction tools (SIFT, PolyPhen-2, BayesDel, Varity, Eve, Alphamissense, REVEL, MetaLR, and CADD) were applied.

### 2.6. Machine Learning Algorithms

After excluding 22/222 clinically defined FH cases with VUS results, the prespecified machine learning (ML) analysis included 200 clinically defined FH cases with binary reportable LP/P variant status (82 positive, 118 with no reportable variant). The candidate predictors were routine referral/sampling variables. Data on triglycerides and VLDL-C were transformed using log1p, and highly correlated continuous variables were pruned on the training set using a prespecified threshold of |r| > 0.90, yielding a final predictor set consisting of age, family history of hyperlipidemia, personal history of cardiac events, lipid-lowering treatment at sampling, LDL-C, HDL-C, and log1p-transformed triglycerides. The procedures for handling missing data, scaling, variable retention decisions, software environment, model-specific tuning strategies, and final selected hyperparameters are summarized in Supplementary Tables S6 and S7. We compared elastic-net logistic regression, random forest, and XGBoost using an approximately 80/20 stratified train/test split, with hyperparameter tuning performed on the training set only. The Simon Broome and DLCN classifications were not used as predictors but were evaluated separately as rule-based comparators. The evaluation of each algorithm was based on the strict rules described by Alay et al. [16].

The primary supervised machine learning-based endpoint was reportable pathogenic/likely pathogenic (LP/P) variant positivity on the targeted panel, rather than the clinical diagnosis of FH per se. Accordingly, the ML analysis should be interpreted as prediction of genetically detectable FH among clinically defined FH cases. FH cases with variants of uncertain significance (VUS) were excluded from the primary binary ML analysis because these VUS results do not provide a definitive deterministic label for supervised learning; however, they were retained as a separate category in the descriptive molecular analyses and figures

### 2.7. Statistical Analysis

Statistical analysis was performed using the Statistical Package for the Social Sciences (SPSS) for Windows, version 30.0, R Studio 4.3, and Python 3.12 programming languages. Descriptive statistics for categorical variables are presented as frequencies and percentages, while numerical variables are expressed as mean ±SD. The normality of the distribution of numerical data was assessed using the Kolmogorov–Smirnov and Shapiro–Wilk tests. Student’s *t* test and Analysis of Variance (ANOVA) were used for analyzing numerical variables with a normal distribution, while the Kruskal–Wallis test and Mann–Whitney U test were applied to data without a normal distribution.

Two-sided *p*-values are reported throughout the paper. Bonferroni adjustment was applied only to post hoc multiple comparisons when applicable; the prespecified two-group comparisons in Table 1 and Table 2 are therefore shown with unadjusted *p*-values, and proportions and odds ratios are reported with 95% confidence intervals where appropriate. Receiver operating characteristic (ROC) analysis was performed, with the area under the curve (AUC), sensitivity, and specificity values calculated, where applicable.

## 3. Results

### 3.1. Descriptive Statistics

Of the 126,097 patients with pre-diagnosed hyperlipidemia referred to Ankara Etlik City Hospital, we only selected familial hyperlipidemia cases. Among the 319 patients referred to the Medical Genetics Department with suspected familial hyperlipidemia, 40 (12.54%) were excluded because molecular analyses were not yet completed at the time of data lock. The final analytic cohort therefore comprised 279 patients, including 222 (79.56%) with an FH phenotype and 57 (20.44%) with an FHTG phenotype. However, cases in which a likely pathogenic or pathogenic variant was identified in one of the hyperlipidemia-related genes, along with a variant in one of the five genes listed above, were retained in the analysis.

### 3.2. Comparison of Hypercholesterolemia and Hypertriglyceridemia

Table 1 summarizes the comparison between patients with familial hypercholesterolemia (FH) and those with familial hypertriglyceridemia (FHTG). As expected, there were statistically significant differences between the two groups, with LDL-C and total cholesterol levels in favor of the FH group, and triglyceride levels in favor of the FHTG group (*p* < 0.05). When the distribution of clinical diagnostic systems across phenotypes was examined, the proportion meeting the Simon Broome criteria was higher in the FHTG group than in the FH group (39/57, 68.4% vs. 116/222, 52.3%; *p* = 0.041). This difference indicated that Simon Broome positivity was approximately twice as likely in FHTG patients (OR ≈ 1.98). Although patients with DLCN positivity was numerically higher in the FHTG group, the difference was not statistically significant (28/57, 49.1% vs. 83/222, 37.4%; *p* = 0.143; OR ≈ 1.62). In contrast, molecular positivity (reportable LP/P variants) was markedly lower in the FHTG group (8/57, 14.0%) and was significantly less frequent compared to the FH group (82/222, 36.9%; *p* = 0.002). Accordingly, the odds of molecular positivity in patients with the FHTG phenotype were lower than in patients with the FH phenotype (OR ≈ 0.28). No statistically significant differences were observed between the two groups in regard to the utilization of lipid-lowering treatment, cardiac history, or familial history (*p* > 0.05).

### 3.3. Comparison of Diagnostic Criteria and Molecular Diagnosis

The comparison of Simon Broome and DLCN scores in patients with familial hypercholesterolemia, based on molecular positivity (reportable LP/P variants), is shown in Table 2. No statistically significant differences were found between patients with a positive or negative molecular diagnosis in terms of Simon Broome and DLCN scores (*p* > 0.05). However, LDL and total cholesterol levels were found to be significantly higher in patients with a positive molecular diagnosis, while HDL levels were found to be higher in those with a negative molecular diagnosis (*p* < 0.05).

### 3.4. Variant Analysis

**Figure 2:**
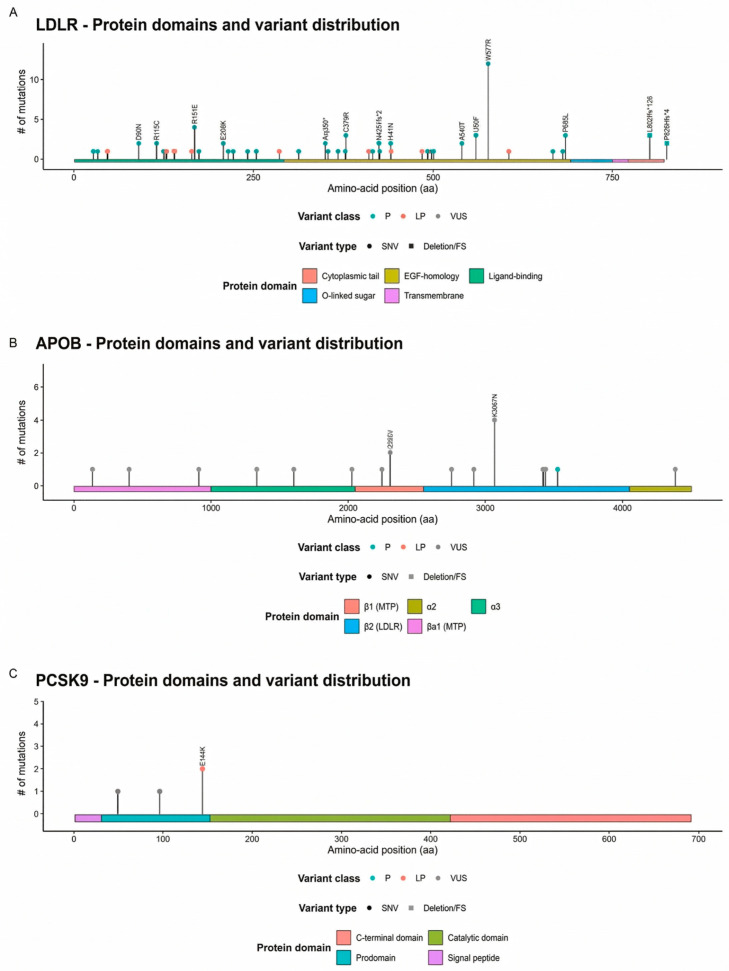
**Protein domain architecture and cohort-specific variant distribution across LDLR, APOB, and PCSK9.** (**A**) *LDLR* variants are distributed across the extracellular portion but are enriched in functionally critical ligand-binding and EGF-homology segments, consistent with receptor binding/folding being the major vulnerability points. A single recurrent hotspot (W577R) dominates the per-site recurrence, and multiple truncating/frameshift events occur in the membrane-proximal/cytoplasmic region, a pattern compatible with loss-of-function mechanisms. (**B**) Compared with *LDLR*, *APOB* harbors fewer cohort variants that are predominantly classified as VUS, with recurrent sites, including I2305V and K3067N, within/near the β2 (LDLR-binding) region; pathogenic calls are rare in this dataset. (**C**) *PCSK9* contributes very few variants overall; a recurrent LP E144K lies near the prodomain–catalytic boundary, whereas the remaining calls are VUS within the prodomain. The lollipop height indicates the number of observations per amino-acid position; color encodes the ACMG/AMP class (P, LP, or VUS) and symbol shape denotes SNV versus deletion/frameshift. Protein domains are annotated as colored bars below each protein.

Figure 2 shows the domain-based distribution of variants in the main hypercholesterolemia-associated genes (LDLR, APOB, and PCSK9). Among these, LDLR variants accounted for the majority of identified variants and were mainly located in the extracellular region, with clustering in the ligand-binding and EGF-homology domains, and W577R was the most frequent variant in this cohort. Both missense and truncating/frameshift variants were observed, including N425Tfs*2, L802Afs*126, and P826Hfs*4. In contrast, APOB harbored few variants, most of which were classified as variants of uncertain significance (VUS); I2305V and K3067N were each observed twice in the β2 (LDLR-binding) region. PCSK9 contributed only a small number of variants, with E144K (LP) being the only notable finding, while all remaining variants were classified as VUS. The variant distributions of the additional four genes are shown in Supplementary Figures S1–S4. Detailed variant analyses for each patient are shown in Supplementary Table S4. Beyond defining the molecular spectrum, we also sought to contextualize these findings geographically across Türkiye, with particular attention to the regional distribution of the novel variants identified in our cohort.

### 3.5. Sociodemographic Analysis of Patients

**Figure 3:**
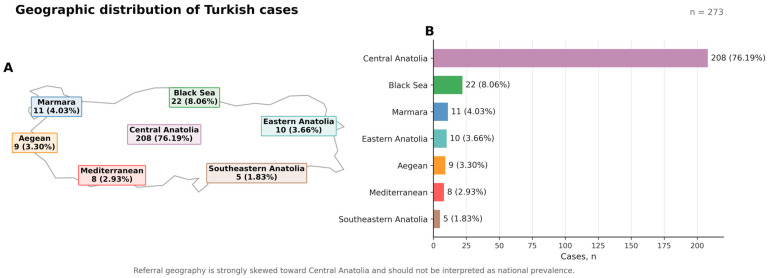
**Geographic distribution of domestic participants in the study cohort across Türkiye.** (**A**,**B**) Data represent the regional distribution of patients born within the national borders (n = 273). Six participants of international origins (2.15%) were excluded to maintain geographic homogeneity and ensure that the findings accurately reflect domestic referral patterns. The analysis of case origin demonstrates marked geographic imbalance, with Central Anatolia contributing the majority of patients within the cohort (208/273, 76.19%), followed by the Black Sea (22/273, 8.06%), Marmara (11/273, 4.03%), Eastern Anatolia (10/273, 3.66%), Aegean (9/273, 3.30%), the Mediterranean (8/273, 2.93%), and Southeastern Anatolia (5/273, 1.83%). The dominance of Central Anatolia indicates a strong center/referral-driven ascertainment pattern, which is important when interpreting regional representativeness and generalizability.

After characterizing the variant spectrum, we next examined the geographic distribution of genetically confirmed cases across the regions represented by our cohort to place the molecular findings in a population-specific context and to explore the regional clustering of novel variants identified within our cohort. The geographic distribution analysis (based on existing regional data) showed that the majority of cases originated from Central Anatolia [Figure 3]. This distribution can be attributed to the fact that our center is located in the Central Anatolia region, consistent with referral/application dynamics. Within this geographic framework, we then performed a focused evaluation of the novel variants identified in the cohort.

### 3.6. Evaluation of Novel Variants

Across the cohort, we identified 13 distinct novel sequence variants (absent from gnomAD) spanning seven genes (*LDLR*, *APOA5*, *CFH*, *PCSK9*, *APOB*, *MC4R*, and *LPL*) and observed in 14 probands (Table 3). The variant classes comprised seven missense variants, two frameshift variants (predicted loss-of-function), two in-frame deletions, one stop-gain, and one canonical splice-site variant. *LDLR* accounted for 7/13 novel variants, distributed across exons 2, 4 (n = 3), 9, 10, and 17, including changes annotated within LDL-receptor class A and class B regions; the recurrent *APOA5* stop-gain c.586G>T p.(Glu196Ter) was detected in two probands (heterozygous and homozygous), both presenting with a hypertriglyceridemic phenotype. Based on the recorded geographic metadata, the probands carrying these novel variants originated from seven provinces—Ankara (n = 8), Çorum (n = 1), Kırıkkale (n = 1), Kırşehir (n = 1), Osmaniye (n = 1), Yozgat (n = 1), and Gümüşhane (n = 1)—and were assigned predominantly to Central Anatolia (13/14) with one proband in the Black Sea region [Table 3]. The detailed evaluation of each novel variant is presented in Supplementary Table S5.

### 3.7. Evaluation of Machine Learning Models and Comparison of Diagnostic Criteria

**Figure 4:**
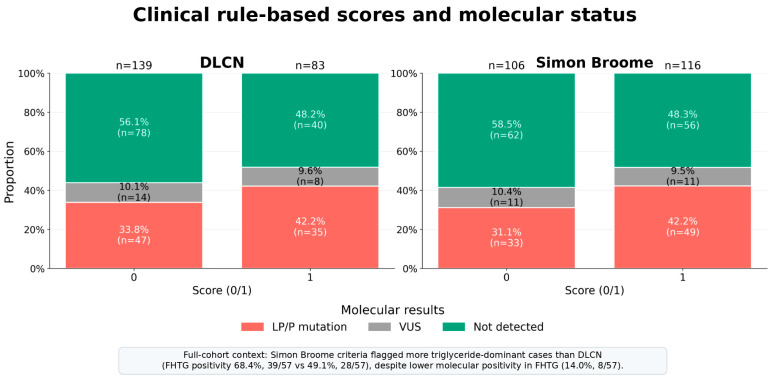
**Comparison of clinical rule-based scores and molecular testing results.** Stacked bars summarize molecular testing outcomes (LP/P variant, VUS, or no variant detected) across binary score strata (0 vs. 1) based on the DLCN and Simon Broome criteria, where score 1 denotes yes/positive and score 0 denotes no/negative, with the sample sizes above the bars. Both systems increase the yield of LP/P variants in individuals with score of 1 (DLCN: 42.2% vs. 33.8%; Simon Broome: 42.2% vs. 31.1%), but a substantial LP/P burden persists in the “low-score” group (~one-third), indicating that rule-based classification alone provides incomplete separation of genetically confirmed cases. VUS proportions remain stable (~9–10%) across strata, implying that dichotomized clinical scoring changes the LP/P yield primarily by shifting “not detected” cases rather than reducing uncertainties.

Notably, 22/222 clinically defined FH cases (9.9%) had VUS results, underscoring that real-world molecular classification is not purely binary; however, because VUS results are non-actionable under current ACMG/AMP guidance and may later be reclassified in either direction, they were retained in the descriptive statistics but excluded from the primary deterministic supervised machine learning analysis [Figure 4]. In the familial hypercholesterolemia (FH) subset of patients with a definitive molecular status (n = 200; 82 positive molecular diagnosis), three supervised models (elastic-net logistic regression, random forest, and XGBoost) were trained to predict molecular positivity (reportable LP/P variant positivity) using an 80/20 stratified train/test split.

**Figure 5:**
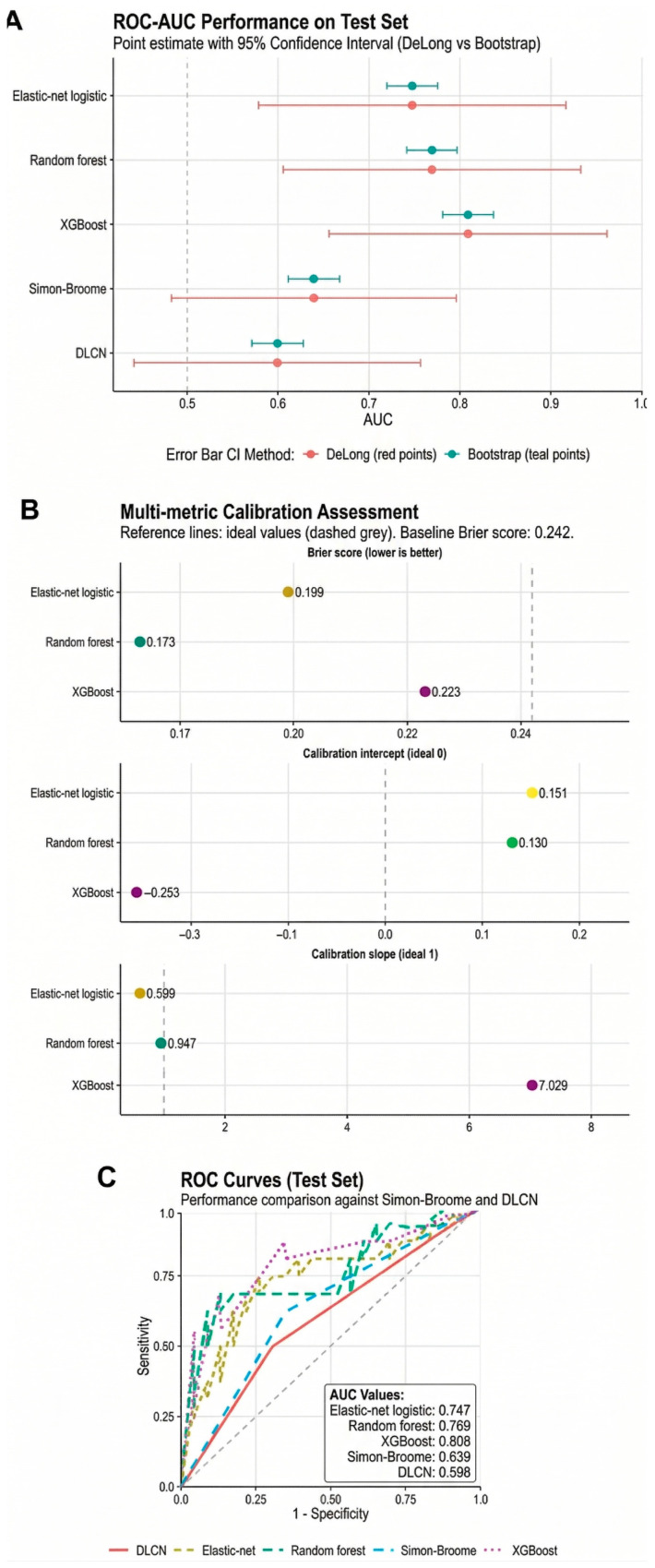
**Test-set discrimination and calibration of machine learning models versus clinical criteria.** (**A**) ROC–AUC point estimates with 95% confidence intervals, computed with DeLong and bootstrap approaches, show clear separation between ML models and rule-based criteria. Discrimination is highest for XGBoost (AUC 0.808), followed by random forest (0.769) and elastic-net logistic regression (0.747), whereas the performance of clinical criteria is lower (Simon Broome 0.639; DLCN 0.598). (**B**) Calibration summaries on the test set (reference lines: intercept = 0, slope = 1; baseline Brier = 0.242) indicate that random forest combines strong discrimination with the best overall calibration (Brier 0.173; slope 0.947), while elastic-net regression shows weaker calibration (slope 0.599). XGBoost, despite having the best AUC, displays pronounced miscalibration (intercept −0.253; slope 7.029), emphasizing that a high AUC does not necessarily translate into well-calibrated probabilities. (**C**) ROC curves confirm consistently improved sensitivity–specificity trade-offs for ML models compared with DLCN and Simon Broome criteria; the inset reports the AUC values. Machine learning analyses were restricted to FH cases with a definitive molecular status (n = 200 after exclusion of VUS) and evaluated using a stratified 80/20 split, yielding 161 training cases and 39 cases in the untouched test set. Given the modest test-set size, AUC differences should be interpreted with caution and not considered stand-alone evidence of model superiority.

On the test set, the ML models trained on routine clinical variables showed higher discrimination for reportable LP/P variant positivity, with AUCs ranging from 0.747 to 0.808 (elastic-net logistic regression: 0.747; random forest: 0.769; XGBoost: 0.808), compared with dichotomized Simon Broome (0.639) and DLCN (0.598) classifications. Importantly, discrimination and probability reliability diverged across models: while XGBoost yielded the highest AUC, random forest produced the most reliable risk estimates, as reflected by the lowest Brier score (0.173) and a calibration slope close to the ideal (0.947). In contrast, XGBoost exhibited marked miscalibration (intercept −0.253; slope 7.029), indicating that its predicted probabilities would require recalibration if used for clinical risk estimation rather than ranking. These findings underscore that good discrimination does not necessarily imply reliable probability estimates and support random forest as the most clinically interpretable and robust model in this cohort [Figure 5]. The detailed results of the machine learning models are presented Supplementary File S1 [Supplementary Figures S5–S8]. Detailed modeling procedures and final selected hyperparameters are shown in Supplementary Tables S6 and S7.

## 4. Discussion

Our study indicates that the two clinical FH diagnostic systems (DLCN and Simon Broome) show limited concordance in identifying patients with reportable FH-associated variants and differ substantially from genetic testing, particularly for patients with both hypercholesterolemia and elevated triglyceride levels. In this setting, rule-based classification appears vulnerable to phenotypic overlap and may misclassify FH-like presentations. Consistent with this, positivity based on Simon Broome criteria was more frequent in patients with triglyceride-dominant profiles than in those with LDL-dominant FH. Importantly, the ML analysis was not designed as a direct FH-versus-FHTG classifier; rather, the FHTG group served as a phenotypic comparator to illustrate overlap and discordance among rule-based criteria. After excluding VUS cases, the prespecified ML analysis was restricted to the clinically defined FH subset and evaluated the models’ discrimination of reportable LP/P variant positivity. Within this subset, the ML models built from routine clinical variables showed better discrimination for reportable LP/P variant positivity than dichotomized rule-based criteria. These findings should not be interpreted as evidence that ML replaces the clinical diagnosis of FH, because a negative genetic test does not exclude FH.

Furthermore, our study introduces several methodological advances relative to prior work. First, the prespecified ML analysis used reportable LP/P variant positivity as the binary molecular endpoint within clinically defined FH, rather than treating molecular status as a universal gold standard for FH diagnosis. Second, the broader cohort analysis included triglyceride-dominant phenotypes to illustrate real-world overlap between FH-like and FHTG-like presentations and to show how rule-based criteria may lose specificity in mixed dyslipidemia presentations. Third, the custom 9-gene panel extended beyond the classical FH genes to include selected genes associated with FHTG and secondary or syndromic hyperlipidemia, thereby broadening the etiologic evaluation in referred patients. Finally, simultaneous evaluation of phenotype overlap, molecular findings, and ML-based prediction was conducted within a single diagnostic workflow for this Turkish tertiary-care cohort.

The significantly higher frequency of positivity based on the Simon Broome criteria in the FHTG group compared with the FH group (68.4% vs. 52.3%) further supports the notion that these criteria may generate false-positive classifications in triglyceride-dominant phenotypes. Because family history and total cholesterol or LDL thresholds constitute the key components in the diagnosis of FH, mixed dyslipidemia patterns—such as type IIb-like phenotypes or conditions accompanied by elevated triglycerides—may be erroneously labeled as “FH-like.” This observation underscores the importance of an initial stratification by the dominant lipid phenotype, whereby marked TG/VLDL elevation characterizes FHTG, while predominant LDL and total cholesterol elevation defines FH. Failure to adequately capture this distinction may result in unnecessary genetic testing or misdirected clinical management.

The presence of elevated triglycerides in patients fulfilling FH diagnostic criteria is also consistent with type IIb hyperlipoproteinemia [26,27,28]. In such cases, pathogenic variants in FH-associated genes may coexist with variants in hypertriglyceridemia-related genes, such as *APOA5* or *LPL* [29]. However, these patients frequently harbor variants of uncertain significance in FH genes, further complicating interpretation. Current Simon Broome and DLCN criteria do not sufficiently account for concomitant hypertriglyceridemia, and, importantly, there are no standardized diagnostic criteria for familial hypertriglyceridemia comparable to those established for FH.

To address potential secondary etiologies, we additionally analyzed *MC4R*, *GNAS*, *GLA*, and *CFH*. This approach enabled the identification of hypercholesterolemia associated with conditions such as obesity-related dyslipidemia, Fabry disease [30], and complement pathway disorders. Although these genes are typically classified under secondary hyperlipidemia, three of five patients harboring pathogenic or likely pathogenic variants in these genes nonetheless fulfilled both the DLCN and Simon Broome diagnostic criteria, highlighting the limited specificity of rule-based criteria in complex or secondary presentations.

Studies in Türkiye show that the mutation burden frequently stems from *LDLR* mutations, with this rate varying between 50% and 95% [23,31]. Furthermore, the frequency of APOB mutations is reported to be lower than that in Europe [31]. A study conducted in Serbia found a high percentage of patients with *LDLR* variants [32], similar to studies conducted in Türkiye. However, in general, the frequency of *LDLR* variants in cases reported in Europe is 60–80%, and the frequency of *APOB* variants is 10–20% [6,33]. The different patterns of variant distribution between Turkish and European populations stem from methodological issues in previous Turkish hyperlipidemia studies, including insufficient sample sizes and patient recruitment from various medical facilities and inadequate implementation of standardized diagnostic criteria [23,31]. In contrast, in our study, we applied more comprehensive and rigorous phenotypic evaluation by at least two medical genetic specialists, along with multiple diagnostic criteria.

An interesting finding of our study is that we detected a pathogenic variant associated with *PCSK9* in only one patient, despite the *LDLR*, *APOB*, and *PCSK9* genes being responsible for 90% of hypercholesterolemia cases [34]. This rate can be considered low when compared to the expected rate in European populations (1–2%) [35] and close to that in Asian populations (<1%) [36]. Surprisingly, the frequency of PCSK9 variants in our cohort was lower than that observed for hypertriglyceridemia-related genes and for genes associated with secondary hypercholesterolemia. Although it is a relatively low rate when compared to the two genes detected at the highest rates in our study, *LDLR* and *APOB*, the use of *PCSK9* inhibitors in treatment is important for the detection of these variants [37].

### 4.1. Machine Learning Algorithms and Diagnostic Criteria

The machine learning algorithms demonstrated better performance than dichotomized Simon Broome and DLCN classifications for predicting reportable LP/P variant positivity within the clinically defined FH subset. The random forest model achieved the most balanced overall performance, combining strong discrimination with superior calibration. These findings are clinically important, as well-calibrated probabilities are essential for effective risk communication and clinical decision-making. Overall, our findings indicate that random forest-based models provide a superior and more comprehensible alternative to conventional diagnostic criteria in familial hypercholesterolemia (FH) [25].

The coexistence of (i) marked phenotypic heterogeneity, including cases with combined FH and triglyceride burden, (ii) discordance between clinical diagnostic criteria and molecular findings, and (iii) geographic and referral clustering observed in this cohort suggests that existing diagnostic frameworks may be influenced by population- and center-specific dynamics. In this context, the development of a Turkey-specific diagnostic or scoring system appears both feasible and clinically relevant.

A practical and scalable next step would be to derive an interpretable triage score for prioritizing clinically suspected FH cases for genetic testing, ideally integrating clinically adjudicated phenotype and molecular findings rather than treating molecular positivity alone as the disease reference standard, while incorporating routinely available clinical variables such as age, LDL-C, total cholesterol, TG/VLDL, HDL-C, treatment status, family history, and history of cardiovascular events. Such a score could be designed to (1) undergo multicenter external validation to assess generalizability; (2) include separate calibration and threshold recommendations for pediatric and adult populations; and (3) provide dual thresholds tailored to distinct clinical use cases, namely screening (high sensitivity) and confirmatory assessment (high specificity).

### 4.2. Novel Variants and Population-Specific Evaluation

The predominance of novel *LDLR* variants in this case series fits with the central role of LDLR dysfunction in LDL-C dominant FH. Their spread across key receptor domains, particularly their clustering on exon 4, supports the clinical relevance of both truncating and non-truncating changes in critical extracellular regions. In parallel, rare variants in triglyceride-related genes highlight the added interpretive complexity in hypertriglyceridemic presentations; for example, the recurrent terminal *APOA5* p.(Glu196Ter) observed in both heterozygous and homozygous states is compatible with a possible dosage effect, while also signifying that terminal truncations can have gene-specific consequences. The identification of a canonical splice-acceptor variant in *CFH* further illustrates the potential of pleiotropic findings on extended panels, reinforcing the need for close phenotype alignment and cautious classification when the lipid profile does not match a gene’s primary disease context. Finally, the geographic concentration of carriers of novel variants in Central Anatolia/Ankara should be interpreted cautiously given the likely referral effects, but it provides a starting point for building more accurate population-specific rare-variant catalogs in familial dyslipidemias.

### 4.3. Strengths and Limitations

This study has several strengths. It integrates clinical classification and targeted molecular testing in a real-world tertiary referral cohort that includes both LDL-dominant and triglyceride-dominant familial dyslipidemia presentations. The combined analysis of Simon Broome and DLCN criteria, molecular findings, and ML-based predictions enabled direct comparison between rule-based and multivariable approaches within the same clinical workflow. Reporting discriminatory power together with calibration metrics and uncertainty estimates also strengthens the interpretation of the ML results.

This study also has important limitations. It was conducted at a single tertiary referral center and is therefore vulnerable to referral clustering and limited external validity. Molecular testing was restricted to the 9-gene targeted panel, so disease-causing variants outside this panel would not have been detected. Pretreatment lipid values were not consistently available; accordingly, both rule-based classifications and ML predictors were based on real-world lipid measurements recorded at referral/sampling, which, in some cases, might have been influenced by ongoing lipid-lowering therapy. In the FH phenotype group, 5 of 222 individuals were receiving lipid-lowering treatment at sampling. Although treatment at sampling was retained as a clinical variable, residual treatment-related confounding could not be excluded. In addition, the primary ML endpoint was reportable LP/P variant positivity on a targeted panel rather than the full clinical diagnosis of FH. Because genetic testing has imperfect sensitivity, some variant-negative patients may still represent clinically true FH. Finally, while exclusion of VUS improved label clarity for supervised machine learning, it reduced direct generalizability to real-world ambiguous molecular results.

## 5. Conclusions

In conclusion, in this cohort, the Simon Broome and DLCN criteria demonstrated limited discriminatory power with respect to both phenotypic differentiation (FH vs. FHTG) and molecular confirmation. Notably, the higher prevalence of FHTG cases identified with the Simon Broome criteria suggests that these criteria may be prone to false-positive classification in triglyceride-predominant phenotypes and therefore may not be sufficient on their own to distinguish FH from FHTG. Taken together, these findings support the use of clinical criteria supplemented by phenotype-based pre-screening and the integration of additional auxiliary approaches, such as molecular testing and multivariable risk modeling (including machine learning-based methods), into the clinical decision-making process in appropriate cases. Machine learning models, particularly XGBoost and random forest, represent a robust starting point for this score-derivation process; however, simplification, interpretability, and external validation are essential prerequisites for successful clinical implementation.

## Figures and Tables

**Table 1:** Comparison of familial hypercholesterolemia and familial hypertriglyceridemia cohorts.

Variable	Familial Hypercholesterolemia (n = 222)	Familial Hypertriglyceridemia (n = 57)	OR (95% CI)	*p*-Value
Age	17.0 [10.0–46.0] (n = 222)	31.0 [12.0–43.0] (n = 57)	—	0.295
Total cholesterol (mg/dL)	252.5 [220.0–300.0] (n = 194)	224.0 [202.5–279.5] (n = 52)	—	0.021
LDL-C (mg/dL)	182.0 [149.0–222.0] (n = 197)	129.0 [71.5–181.5] (n = 51)	—	<0.001
HDL-C (mg/dL)	52.0 [43.0–62.0] (n = 164)	33.0 [27.0–40.0] (n = 45)	—	<0.001
VLDL-C (mg/dL)	18.5 [14.8–27.2] (n = 80)	103.0 [55.2–184.0] (n = 30)	—	<0.001
Triglycerides (mg/dL)	106.0 [73.5–139.5] (n = 183)	458.0 [297.0–912.0] (n = 57)	—	<0.001
Family history of hyperlipidemia	128/221 (57.9%; 95% CI 51.3–64.2)	29/57 (50.9%; 95% CI 38.3–63.4)	0.75 (0.42–1.35)	0.42
Cardiac event history	32/222 (14.4%; 95% CI 10.4–19.6)	5/57 (8.8%; 95% CI 3.8–18.9)	0.57 (0.21–1.54)	0.367
Lipid-lowering treatment at sampling	5/222 (2.3%; 95% CI 1.0–5.2)	0/57 (0.0%; 95% CI 0.0–6.3)	0.34 (0.02–6.31) †	0.587
Simon Broome criteria (SB) met	116/222 (52.3%; 95% CI 45.7–58.7)	39/57 (68.4%; 95% CI 55.5–79.0)	1.98 (1.07–3.67)	0.041
DLCN score positive	83/222 (37.4%; 95% CI 31.3–43.9)	28/57 (49.1%; 95% CI 36.6–61.7)	1.62 (0.90–2.91)	0.143
Molecular diagnosis positive *	82/222 (36.9%; 95% CI 30.9–43.5)	8/57 (14.0%; 95% CI 7.3–25.3)	0.28 (0.13–0.62)	0.002

Continuous variables are reported as median [IQR] with the number of non-missing observations in parentheses. Categorical variables are reported as n/N (%; 95% CI). *p*-values are two-sided and unadjusted for these prespecified two-group comparisons (Mann–Whitney U test for continuous variables; chi-square test or Fisher’s exact test for categorical variables, as appropriate). ORs are provided for categorical variables. † For rows with a zero cell, the OR and 95% CI were estimated using Haldane–Anscombe continuity correction. * Cases with detected LP/P variants.

**Table 2:** Comparison of familial hypercholesterolemia patients by molecular diagnosis status.

Variable	LP/P Variant Detected (n = 82) *	No Reportable Variant Detected (n = 118) *	OR (95% CI)	*p*-Value
Age	14.5 [9.0–37.2] (n = 82)	26.0 [11.0–48.0] (n = 118)	—	0.091
Total cholesterol (mg/dL)	261.5 [242.2–316.2] (n = 76)	236.0 [214.0–288.0] (n = 97)	—	<0.001
LDL-C (mg/dL)	202.5 [180.2–247.0] (n = 76)	162.0 [141.0–212.0] (n = 101)	—	<0.001
HDL-C (mg/dL)	50.0 [41.0–59.0] (n = 65)	56.5 [46.0–65.0] (n = 82)	—	0.019
VLDL-C (mg/dL)	17.0 [12.2–24.0] (n = 34)	20.5 [17.0–29.8] (n = 36)	—	0.059
Triglycerides (mg/dL)	90.0 [67.0–120.0] (n = 73)	116.0 [84.5–149.0] (n = 91)	—	0.022
Family history of hyperlipidemia	55/81 (67.9%; 95% CI 57.1–77.1)	64/118 (54.2%; 95% CI 45.3–63.0)	1.78 (0.99–3.22)	0.074
Cardiac event history	12/82 (14.6%; 95% CI 8.6–23.9)	15/118 (12.7%; 95% CI 7.9–19.9)	1.18 (0.52–2.67)	0.856
Lipid-lowering treatment at sampling	5/82 (6.1%; 95% CI 2.6–13.5)	0/118 (0.0%; 95% CI 0.0–3.2)	16.82 (0.92–308.51) †	0.011
Simon Broome criteria (SB) met	49/82 (59.8%; 95% CI 48.9–69.7)	56/118 (47.5%; 95% CI 38.7–56.4)	1.64 (0.93–2.91)	0.117
DLCN score positive	35/82 (42.7%; 95% CI 32.5–53.5)	40/118 (33.9%; 95% CI 26.0–42.8)	1.45 (0.81–2.59)	0.265

Analysis restricted to familial hypercholesterolemia cases with molecular diagnosis coded as yes or no; cases with VUS results were excluded from this primary binary analysis (n = 22). Continuous variables are reported as median [IQR] with the number of non-missing observations in parentheses. Categorical variables are reported as n/N (%; 95% CI). *p*-values are two-sided and unadjusted for these prespecified two-group comparisons (Mann–Whitney U test for continuous variables; chi-square test or Fisher’s exact test for categorical variables, as appropriate). ORs are provided for categorical variables. † For rows with a zero cell, the OR and 95% CI were estimated using Haldane–Anscombe continuity correction. * Positive molecular diagnosis = LP/P variant detected; negative molecular diagnosis = no reportable variant detected.

**Table 3:** Novel variants identified in the cohort.

Gene	Variant (HGVS)	Variant Class	Zygosity/Proband	Key Phenotype	gnomAD	Key Notes
*APOA5*	c.586G>T; p.(Glu196Ter)	Stop-gain	Het (52F); Hom (29F)	High TG; family history in 52F	Not reported	Terminal exon; HI tolerance suggested; no prior identical pathogenic report
*LDLR*	c.2477_2493del; p.(Pro826HisfsTer4)	Frameshift (predicted LoF)	Het; 18F	High total cholesterol; parental hyperlipidemia	Not reported	Exon 17; predicted NMD; ClinGen HI; LoF variants predominantly pathogenic
*LDLR*	c.372del; p.(Gln125SerfsTer81)	Frameshift (predicted LoF)	Het; 27F	Lipid profile unavailable; maternal hyperlipidemia	Not reported	Exon 4; regional pathogenic reports; ClinGen HI supports LoF relevance
*LDLR*	c.1496C>T; p.(Ser499Phe)	Missense	Het; 9F	High total and LDL-C; early cardiac disease in family	Not reported	In silico deleterious; other pathogenic variants at same codon reported
*LDLR*	c.140A>G; p.(Asp47Gly)	Missense	Hom; 8F	High total cholesterol; LDL-C predominant	Not reported	In silico unanimously deleterious; variants at same residue reported as pathogenic/VUS
*LDLR*	c.386A>T; p.(Asp129Val)	Missense	Het; 31F	High total cholesterol; LDL-C predominant; family history positive	Not reported	In silico unanimously deleterious; variants at same residue reported as pathogenic/VUS
*LDLR*	c.418_426del; p.(Glu140_Ser142del)	In-frame deletion	Het; 59F	High total cholesterol; LDL-C predominant; family history positive	Not reported	Exon 4; regional pathogenic reports; LDLR small indels frequently pathogenic
*CFH*	c.2237-1G>T (splice acceptor)	Canonical splice-site	Het; 42M	High total cholesterol; LDL-C predominant	Not reported	SpliceAI 0.94; CFH LoF constrained (pLI 1); classified VUS in this context
*PCSK9*	c.145G>A; p.(Glu49Lys)	Missense	Het; 15M	High total cholesterol; LDL-C predominant	Not reported	Low missense constraint; in silico not supportive; no prior pathogenic report at residue
*APOB*	c.10256A>G; p.(Lys3419Arg)	Missense	Het; 45M	High total cholesterol; LDL-C predominant; family cardiac history	Not reported	Low missense constraint; in silico not supportive; no prior pathogenic report at residue
*MC4R*	c.990C>A; p.(Ser330Arg)	Missense	Het; 27F	High TG; elevated total cholesterol	Not reported	In silico not supportive; no prior pathogenic report at residue
*LPL*	c.226T>A; p.(Phe76Ile)	Missense	Het; 35M	High TG; elevated total cholesterol	Not reported	In silico suggests deleterious; LPL missense variants often pathogenic; no prior pathogenic report at residue
*LDLR*	c.1231_1242del; p.(Lys411_Leu414del)	In-frame deletion	Het; 8F	High total cholesterol; prominent LDL-C elevation	Not reported	Exon 9; regional pathogenic reports; LDLR in-frame indels frequently pathogenic

**Note:** In the individual carrying CFH c.2237-1G>T, a heterozygous duplication spanning APOB exons 1–28 was also suspected on CNV analysis.

## Data Availability

The datasets generated and/or analyzed during the current study are not publicly available due to patient confidentiality and institutional restrictions, but they are available from the corresponding author upon reasonable request and subject to ethics approval where applicable.

## Supplementary Materials

The following supporting information can be downloaded at: https://www.mdpi.com/article/10.3390/life16040633/s1, Figure S1: Distribution of variants across the APOA5-encoded protein and its functional domains; Figure S2: Distribution of variants across the MC4R-encoded protein; Figure S3: Distribution of variants across the LPL-encoded protein and its domains; Figure S4: Distribution of variants across the GNAS-encoded protein and its domains; Figure S5: Correlation-based heatmap for feature selection for machine learning-based classification systems (Pearson correlation was used). Age, family history of hyperlipidemia, cardiac history, treatment, LDL, HDL, and TG_log were included as features for machine learning models; Figure S6: LDL vs. total cholesterol multicollinearity comparison; Figure S7: TG vs. VLDL multicollinearity comparison; Figure S8: Learning curves according to training set; Table S1: MEDPED criteria; Table S2: Dutch Lipid Clinic Network (DLCN) criteria; Table S3: Simon Broome criteria; Table S4: Comprehensive evaluation of patients harboring genetic variants; Table S5: Detailed evaluation of novel variants. Case-based descriptions and variant-level evidence summaries for novel sequence variants detected in genes associated with lipid metabolism and familial dyslipidemias. Variant nomenclature follows the HGVS recommendations.

## Abbreviations

ACMG	American College of Medical Genetics and Genomics
AHA/ACC	American Heart Association/American College of Cardiology
AMP	Association for Molecular Pathology
ANNOVAR	ANNOtate VARiation
ASCVD	atherosclerotic cardiovascular disease
AUC	area under the curve
CI	confidence interval
ClinGen	Clinical Genome Resource
ClinVar	Clinical Variation database
CNV	copy-number variant
dbNSFP	database for nonsynonymous SNPs’ functional predictions
DLCN	Dutch Lipid Clinic Network
EDTA	ethylenediaminetetraacetic acid
EGF	epidermal growth factor
ESP	NHLBI Exome Sequencing Project
ExAC	Exome Aggregation Consortium
FH	familial hypercholesterolemia
FHTG	familial hypertriglyceridemia
GATK	Genome Analysis Toolkit
gnomAD	Genome Aggregation Database
HDL-C	high-density lipoprotein cholesterol
HGMD	Human Gene Mutation Database
HGVS	Human Genome Variation Society
HI	haploinsufficiency
IQR	interquartile range
LDL-C	low-density lipoprotein cholesterol
LoF	loss-of-function
LOVD	Leiden Open Variation Database
MEDPED	Make Early Diagnosis to Prevent Early Death
ML	machine learning
MQ	mapping quality
NGS	next-generation sequencing
NMD	nonsense-mediated decay
OR	odds ratio
P	pathogenic
pLI	probability of loss-of-function intolerance
PolyPhen-2	Polymorphism Phenotyping v2
Q	base quality score
REVEL	Rare Exome Variant Ensemble Learner
ROC	receiver operating characteristic
SB	Simon Broome
SBS	sequencing by synthesis
SD	standard deviation
SIFT	Sorting Intolerant From Tolerant
SNV	single nucleotide variant
TG	triglycerides
VLDL-C	very-low-density lipoprotein cholesterol
VUS	variant of uncertain significance
XGBoost	Extreme Gradient Boosting
