# Supplementary material for: Integrated Clinical, Molecular, and Machine Learning Assessment of Familial Hypercholesterolemia
Source: Life (Basel). 2026 Apr 9;16(4):633. doi: 10.3390/life16040633 (PMC13117847; doi:10.3390/life16040633)
Supplement: Supplementary file 1 [file life-16-00633-s001.zip › life-4195279-supplementary.pdf]

Supplementary Files

A. **Abstract of Supplementary Files**

1. **Legend of Supplementary Figures**

Supplementary Figure S1. Distribution of variants across the *APOA5*-encoded protein and its functional domains

Supplementary Figure S2. Distribution of variants across the *MC4R*-encoded protein

Supplementary Figure S3. Distribution of variants across the *LPL*-encoded protein and its domains

Supplementary Figure S4. Distribution of variants across the *GNAS*-encoded protein and its domains

Supplementary Figure S5. Correlation-based heatmap for feature selection for the machine learning classification system (Pearson correlation was used). Age, family history, cardiac history, treatment, LDL, HDL, and TG_log were included as features for the machine learning models.

Supplementary Figure S6. LDL vs total cholesterol multicollinearity comparison

Supplementary Figure S7. TG vs VLDL multicollinearity comparison

Supplementary Figure S8. Learning curves according to training set

2. **Supplementary Tables**

Supplementary Table S1. MEDPED Criteria

Supplementary Table S2. Dutch Lipid Clinic Network (DLCN) Criteria

Supplementary Table S3. Simon-Broome Criteria

Supplementary Table S4. Comprehensive evaluation of patients harboring genetic variants

Supplementary Table S5. Detailed evaluation of novel variants. Case-based descriptions and variant-level evidence summaries for novel sequence variants detected in genes associated with lipid metabolism and familial dyslipidemias. Variant nomenclature follows HGVS recommendations.

Supplementary Figures

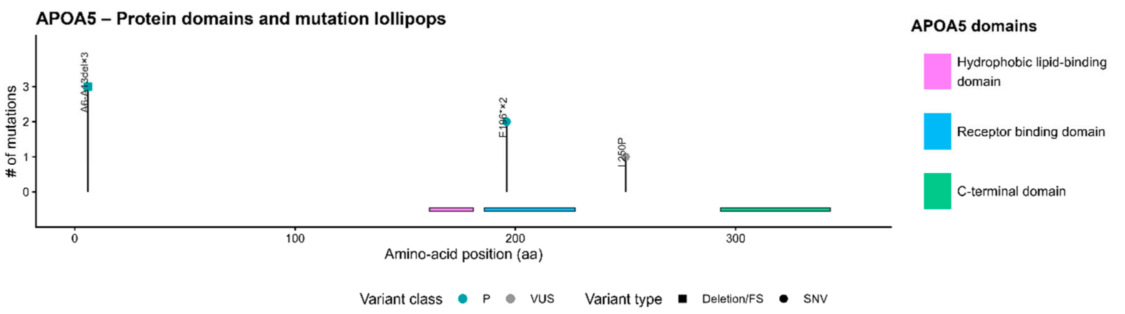

Supplementary Figure S1. Distribution of variants across the APOA5-encoded protein and its functional domains

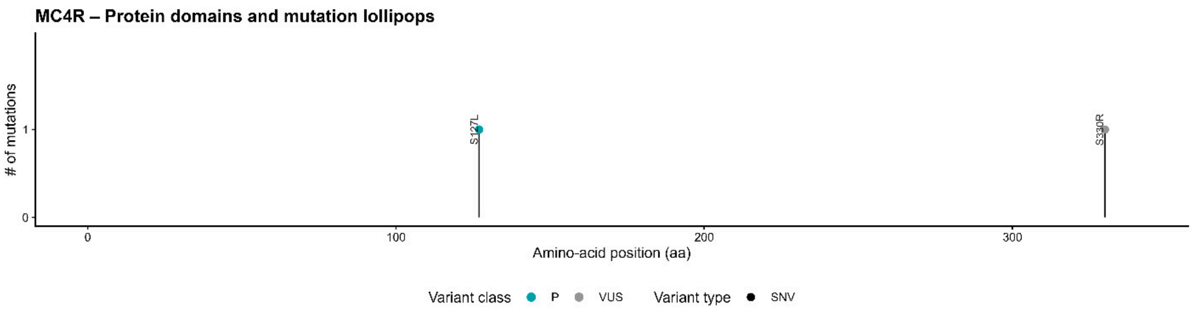

Supplementary Figure S2. Distribution of variants across the MC4R-encoded protein

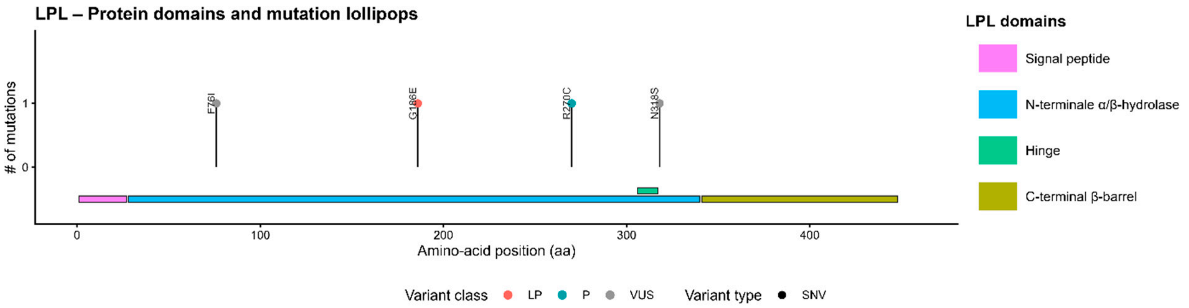

Supplementary Figure S3. Distribution of variants across the LPL-encoded protein and its domains

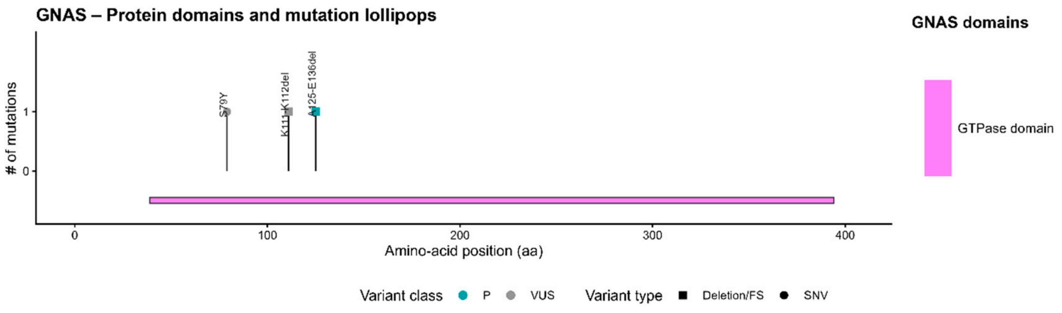

Supplementary Figure S4. Distribution of variants across the GNAS-encoded protein and its domains

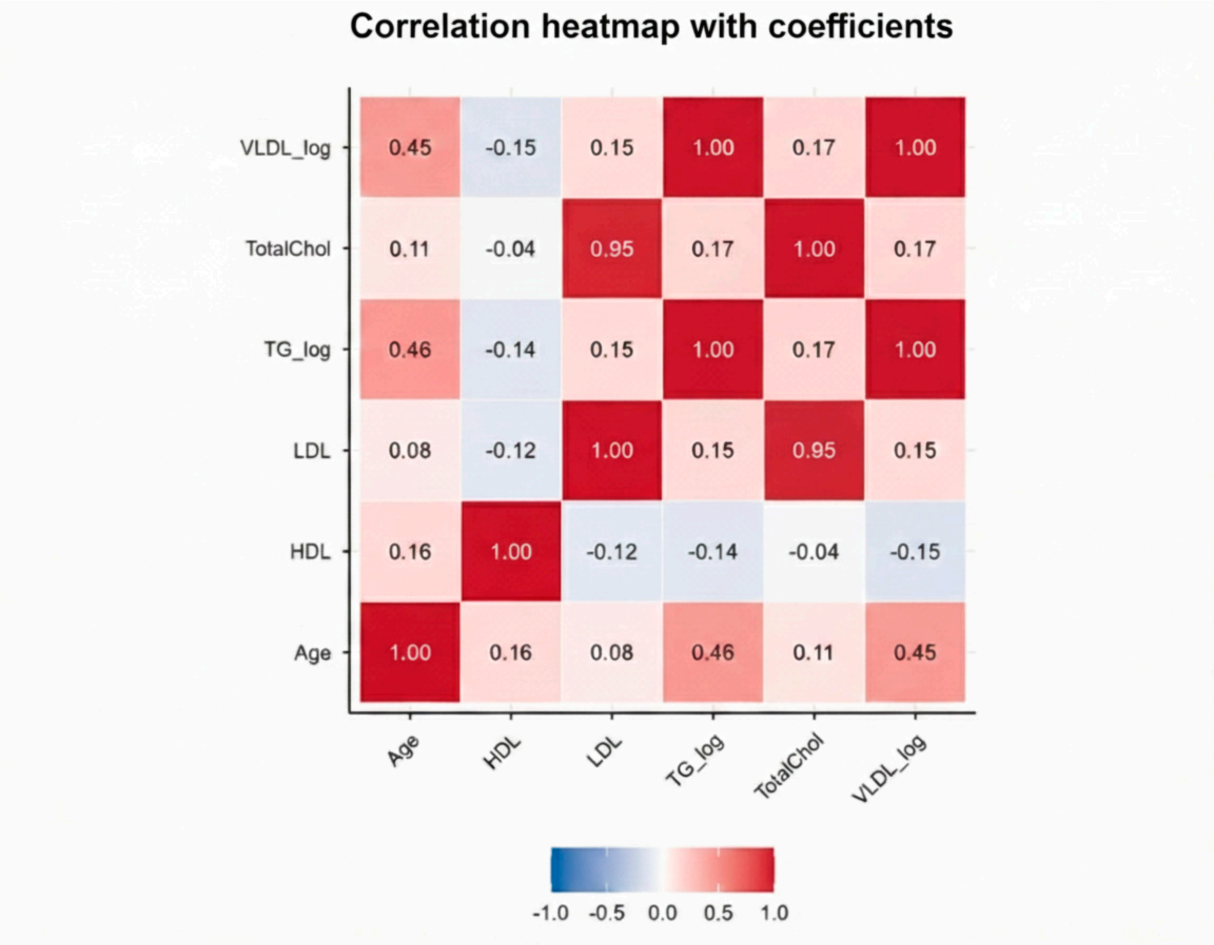

Supplementary Figure S5. Correlation-based heatmap for feature selection for the machine learning classification system (Pearson correlation was used). Age, family history, cardiac history, treatment, LDL, HDL, and TG_log were included as features for the machine learning models.

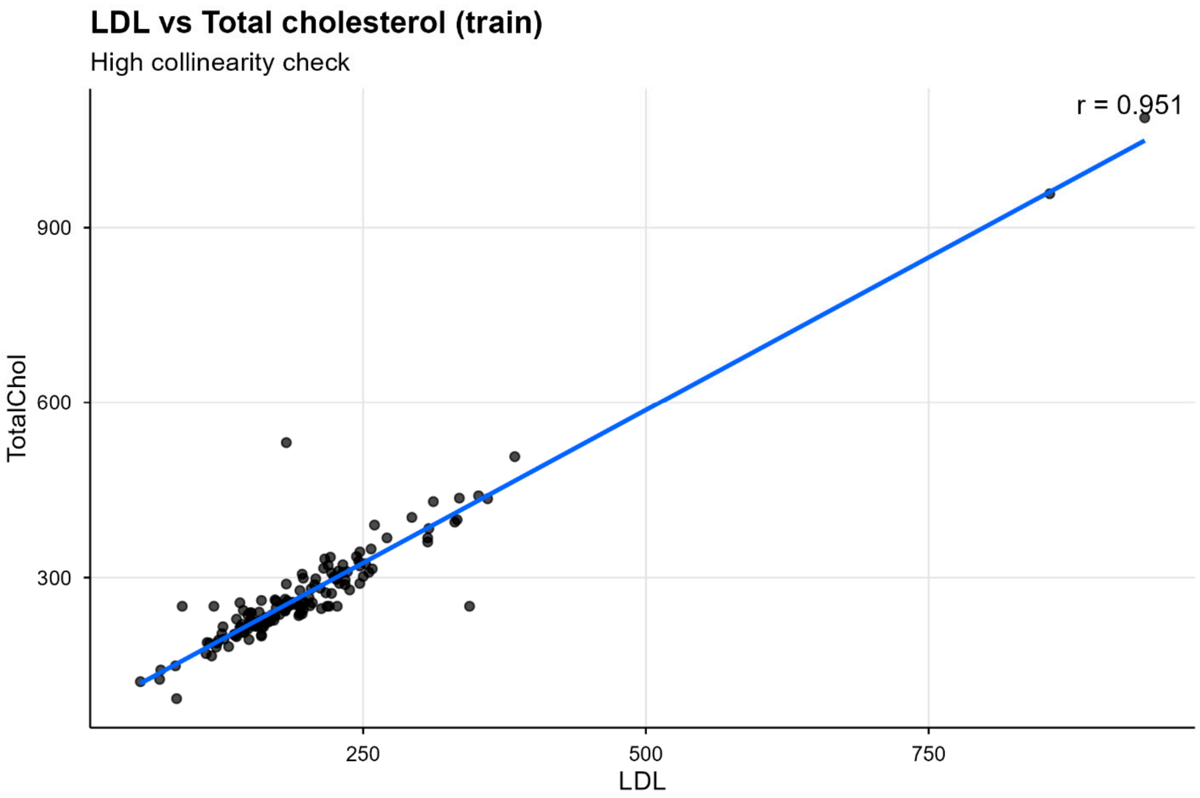

Supplementary Figure S6. LDL vs total cholesterol multicollinearity comparison

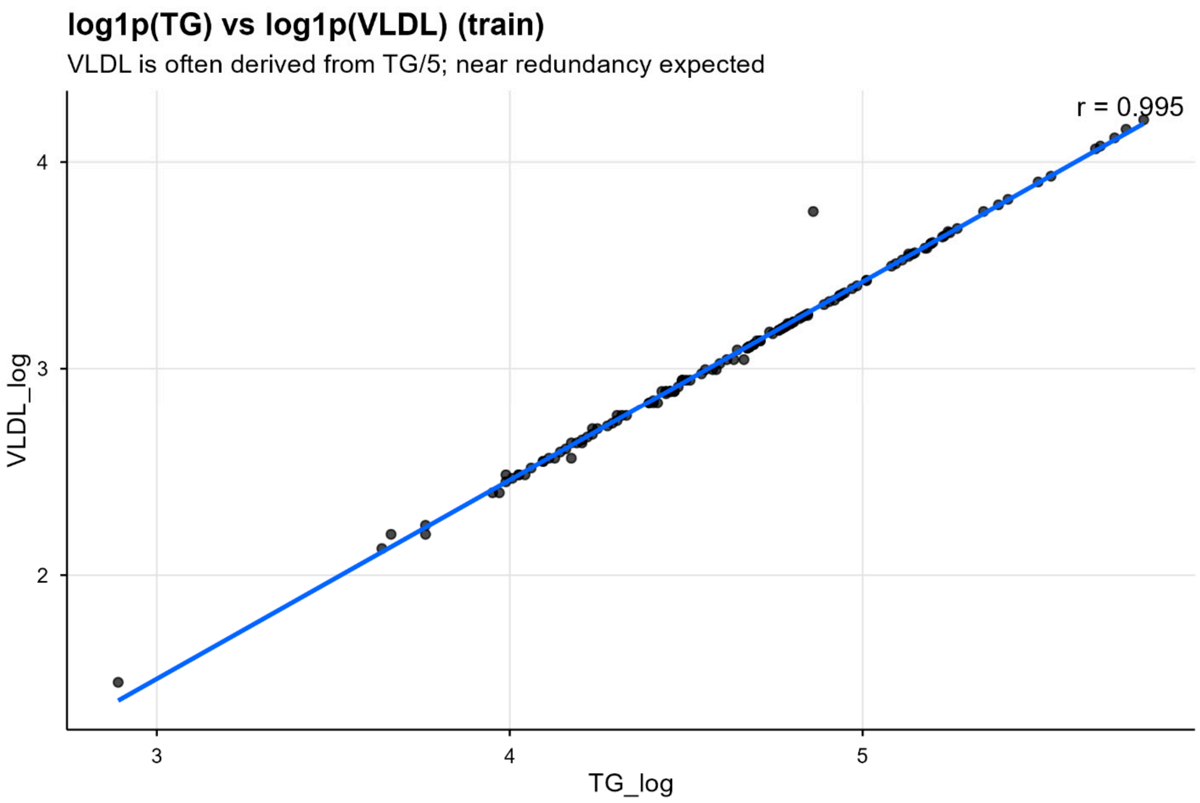

Supplementary Figure S7. TG vs VLDL multicollinearity comparison

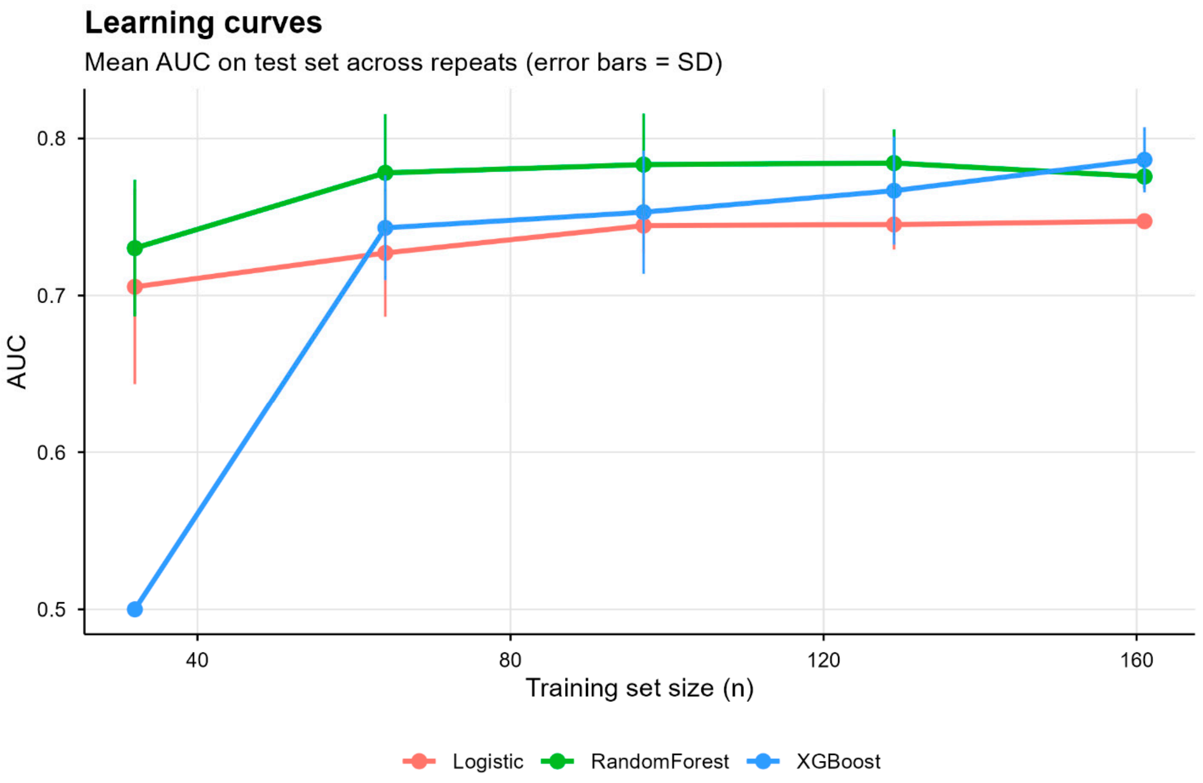

Supplementary Figure S8. Learning curves according to training set

Supplementary Tables

Supplementary Table S1 MEDPED Criteria. Age-specific total cholesterol and LDL-C cut points for FH according to relationship to an affected relative.

Age (years)	First-degree relative TC (LDL-C), mg/dL	Second-degree relative TC (LDL-C), mg/dL	Third-degree relative TC (LDL-C), mg/dL	General population TC (LDL-C), mg/dL
<20	220 (155)	230 (165)	240 (170)	270 (200)
20–29	240 (170)	250 (180)	260 (185)	290 (220)
30–39	270 (190)	280 (200)	290 (210)	340 (240)
≥40	290 (205)	300 (215)	310 (225)	360 (260)

Note: MEDPED = Make Early Diagnosis to Prevent Early Death. These cutoffs have been reported as achieving approximately 98% specificity, with sensitivity varying by relationship category. Source: NCBI Bookshelf, Appendix D / AHRQ evidence review.

Supplementary Table S2. Dutch Lipid Clinic Network (DLCN) Criteria. Point-based diagnostic criteria for clinical classification of FH.

**Domain**	**Criterion**	**Points**
Family history	First-degree relative with premature cardiovascular disease or LDL-C >95th percentile	1
Family history	First-degree relative with tendinous xanthoma and/or corneal arcus, or child (<18 years) with LDL-C >95th percentile	2
Clinical history	Personal history of premature coronary artery disease	2
Clinical history	Personal history of premature peripheral or cerebrovascular disease	1
Physical examination	Corneal arcus in a patient aged <45 years	4
Physical examination	Tendon xanthoma	6
LDL-C level	LDL-C 155–189 mg/dL	1
LDL-C level	LDL-C 190–249 mg/dL	3
LDL-C level	LDL-C 250–329 mg/dL	5
LDL-C level	LDL-C ≥330 mg/dL	8
DNA analysis	Functional mutation in LDLR, APOB, or PCSK9 / causative FH mutation	8
Interpretation	Definite FH	≥8 total points
Interpretation	Probable FH	6–7 total points
Interpretation	Possible FH	3–5 total points
Interpretation	Unlikely FH	<3 total points

Note: Some published summaries compress the original WHO/Dutch criteria into point-level statements. This table follows the widely used clinical summary reproduced in NCBI Bookshelf. Source: NCBI Bookshelf, Appendix D; WHO consultation-based Dutch criteria summary.

Supplementary Table S3. Simon–Broome Criteria. Diagnostic criteria for definite, probable, and possible FH according to the Simon–Broome Register framework.

**Category**	**Required criteria**
Cholesterol threshold	Adults: total cholesterol >290 mg/dL or LDL-C >190 mg/dL; children (<16 years): total cholesterol >260 mg/dL or LDL-C >155 mg/dL
Definite FH	Cholesterol threshold AND either (1) a DNA mutation in LDLR, APOB, or PCSK9, or (2) tendon xanthomas in the patient or in a first- or second-degree relative
Possible FH	Cholesterol threshold AND either (1) family history of myocardial infarction before age 60 years in a first-degree relative or before age 50 years in a second-degree relative, OR (2) family history of total cholesterol >290 mg/dL in a first- or second-degree relative

Note: In several modern summaries, the tendon xanthoma criterion is listed under "definite FH," whereas family-history-only combinations are classified as "possible FH." Source: NCBI Bookshelf summary of the Simon Broome Register criteria.

Supplementary Table S4. Comprehensive evaluation of patients harboring genetic variants

Patient ID	Age	Transcript (NM_)	Variant (HGVS)	Zygosity	ACMG Classification	SBS	DCLN Score	Geographic origin
P1	2	NM_000516.7	GNAS c.374_409del p.(Ala125_Glu136del)	Heterozygous	P	No	No	Ankara
P2	2	NM_000527.5	LDLR c.520G>T p.(Glu174Ter)	Heterozygous	P	Yes	No	Ankara
P3	3	NM_000527.5	LDLR c.1135T>C p.(Cys379Arg)	Heterozygous	P	Yes	Yes	Afyon
P4	4	NM_000527.5	LDLR c.1135T>C p.(Cys379Arg)	Heterozygous	P	Yes	Yes	Afyon
P5	5	NM_000384.3	APOB c.10580G>A p.(Arg3527Gln)	Heterozygous	P	No	No	Ankara
P6	5	NM_000527.5	LDLR c.1618G>A p.(Ala540Thr)	Heterozygous	P	Yes	No	Corum
P7	5	NM_000527.5	LDLR c.1999T>C p.(Cys667Arg)	Heterozygous	P	Yes	Yes	Eskisehir
P8	5	NM_000527.5	LDLR c.2312-3C>A	Heterozygous	P	Yes	Yes	Ankara
P9	6	NM_000527.5	LDLR c.268G>A p.(Asp90Asn)	Heterozygous	P	Yes	No	Ankara
P10	6	NM_000384.3	APOB c.4808A>T p.(Gln1603Leu)	Heterozygous	VUS	No	No	Istanbul
P11	6	NM_000384.3	APOB c.9201G>T p.(Lys3067Asn)	Heterozygous	VUS	Yes	Yes	United States
P12	6	NM_016592.5	GNAS c.236C>A p.(Ser79Tyr)	Heterozygous	VUS	No	No	Ankara
P13	7	NM_000527.5	LDLR c.1478_1479del p.(Ser493CysfsTer42)	Heterozygous	P	Yes	Yes	Ankara
P14	7	NM_000527.5	LDLR c.622G>A p.(Glu208Lys)	Homozygous	P	No	No	Samsun
P15	7	NM_000527.5	LDLR c.643C>A p.(Arg215Ser)	Heterozygous	P	No	No	Ankara
P16	8	NM_000527.5	LDLR c.1231_1242del p.(Lys411_Leu414del)	Heterozygous	LP	No	No	Ankara
P17	8	NM_000527.5	LDLR c.140A>G p.(Asp47Gly)	Homozygous	LP	No	No	Iraq
P18	8	NM_000527.5	LDLR c.858C>A p.(Ser286Arg)	Heterozygous	LP	Yes	No	Ankara
P19	8	NM_000527.5	LDLR c.1729T >C p.(Trp577Arg)	Heterozygous	P	No	No	Ankara
P20	8	NM_000527.5	LDLR c.268G>A p.(Asp90Asn)	Heterozygous	P	Yes	No	Manisa
P21	8	NM_000384.3	APOB c.1205A>G p.(His402Arg)	Heterozygous	VUS	Yes	Yes	Ankara
P22	8	NM_000384.3	APOB c.8746G>C p.(Ala2916Pro)	Heterozygous	VUS	Yes	No	Ankara
P23	8	NM_174936.4	PCSK9 c.430G>A p.(Glu144Lys)	Heterozygous	VUS	No	No	Ankara
P24	9	NM_000527.5	LDLR c.1454A>G p.(His485Arg)	Heterozygous	LP	Yes	No	Cankiri
P25	9	NM_000527.5	LDLR c.1496C>T p.(Ser499Phe)	Heterozygous	LP	Yes	Yes	Ankara
P26	9	NM_000527.5	LDLR c.1135T>C p.(Cys379Arg)	Heterozygous	P	Yes	Yes	Afyon
P27	9	NM_000527.5	LDLR c.2403_2406del p.(Leu802AlafsTer126)	Heterozygous	P	Yes	No	Kastamonu
P28	9	NM_000527.5	LDLR c.504C>A p.(Asp168Glu)	Heterozygous	P	Yes	Yes	Ankara
P29	9	NM_000527.5	LDLR c.97C>T p.(Gln33Ter)	Heterozygous	P	No	No	Sakarya
P30	10	NM_000527.5	LDLR c.1322T>A p.(Ile441Asn)	Heterozygous	LP	Yes	No	Ankara

Patient ID	Age	Transcript (NM_)	Variant (HGVS)	Zygosity	ACMG Classification	SBS	DCLN Score	Geographic origin
P31	10	NM_000527.5	LDLR c.1729T >C p.(Trp577Arg)	Heterozygous	P	Yes	Yes	Ankara
P32	10	NM_000237.3	LPL c.808C>T p.(Arg270Cys)	Heterozygous	P	No	No	Nigde
P33	10	NM_001371904.1/ NM_000527.5	APOA5 c.16_39del p.Ala6_Ala13del / LDLR c.185C>T p.(Thr62Met)	Homozygous/ Heterozygous	P/ LP	Yes	No	Ankara
P34	10	NM_000527.5	APOA5 c.749T>C p.(Leu250Pro)	Heterozygous	VUS	Yes	Yes	Ankara
P35	10	NM_000384.3	APOB c.13151T>C p.(Leu4384Pro)	Heterozygous	VUS	Yes	Yes	Ankara
P36	11	NM_000527.5	LDLR c.1324T>C p.(Tyr442His)	Heterozygous	LP	No	No	Ankara
P37	11	NM_000527.5	LDLR c.1132C>T p.(Gln378Ter)	Heterozygous	P	No	Yes	Ankara
P38	11	NM_000527.5	LDLR c.1729T >C p.(Trp577Arg)	Heterozygous	P	No	No	Ankara
P39	11	NM_005912.3	MC4R c.380C>T p.(Ser127Leu)	Heterozygous	P	No	No	Ankara
P40	12	NM_000527.5	LDLR c.1729T >C p.(Trp577Arg)	Heterozygous	P	Yes	No	Ankara
P41	12	NM_000527.5	LDLR c.2403_2406del p.(Leu802AlafsTer126)	Heterozygous	P	No	No	Kastamonu
P42	12	NM_000384.3	APOB c.3997C>G p.(Arg1333Gly)	Heterozygous	VUS	Yes	Yes	Ankara
P43	12	NM_174936.4	PCSK9 c.430G>A p.(Glu144Lys)	Heterozygous	VUS	No	No	Ankara
P44	13	NM_000527.5	LDLR c.2041T>A p.(Cys681Ser)	Heterozygous	P	No	No	Ankara
P45	13	NM_000527.5	LDLR c.378del p.(Phe126LeufsTer80)	Heterozygous	P	Yes	No	Ankara
P46	13	NM_000527.5	LDLR c.724C>T p.(Gln242Ter)	Heterozygous	P	Yes	Yes	Ankara
P47	14	NM_001371904.1	APOA5 c.16_39del p.(Ala6_Ala13del)	Homozygous	LP	Yes	No	Ankara
P48	14	NM_000527.5	LDLR c.491T>C p.(Leu164Pro)	Heterozygous	LP	No	No	Corum
P49	14	NM_000527.5	LDLR c.1277T>G p.(Leu426Arg)	Heterozygous	P	No	No	Ankara
P50	14	NM_000527.5	LDLR c.1322T>A p.(Ile441Asn)	Heterozygous	P	No	No	Ankara
P51	14	NM_000527.5	LDLR c.1729T >C p.(Trp577Arg)	Heterozygous	P	No	No	Van
P52	14	NM_000527.5	LDLR c.1729T >C p.(Trp577Arg)	Heterozygous	P	Yes	No	Ankara
P53	14	NM_000527.5	LDLR c.2054C>T p.(Pro685Leu)	Heterozygous	P	Yes	Yes	Ankara
P54	14	NM_000527.5	LDLR c.504C>A p.(Asp168Glu)	Heterozygous	P	Yes	Yes	Ankara
P55	15	NM_000527.5	LDLR c.1816G>A p.(Ala606Thr) / LDLR c.556G>C p.(Gly186Arg)	Heterozygous / Heterozygous	LP/ VUS	Yes	No	Ankara
P56	15	NM_000527.5	LDLR c.1586+5G>A	Heterozygous	P	No	No	Ankara
P57	15	NM_000527.5	LDLR c.1729T>C p.(Trp577Arg)	Heterozygous	P	Yes	Yes	Ankara
P58	15	NM_000527.5	LDLR c.2054C>T p.(Pro685Leu)	Heterozygous	P	Yes	No	Yozgat

Patient ID	Age	Transcript (NM_)	Variant (HGVS)	Zygosity	ACMG Classification	SBS	DCLN Score	Geographic origin
P59	15	NM_000527.5	LDLR c.81C>G p.(Cys27Trp)	Heterozygous	P	No	No	Duzce
P60	15	NM_000384.3	APOB c.6079C>A p.(Pro2027Thr)	Heterozygous	VUS	Yes	Yes	Ankara
P61	15	NM_174936.4	PCSK9 c.145G>A p.(Glu49Lys)	Heterozygous	VUS	No	No	Ankara
P62	15	NM_174936.4	PCSK9 c.287G>A p.(Arg96HisS)	Heterozygous	VUS	No	No	Tokat
P63	16	NM_000527.5	LDLR c.343C>T p.(Arg115Cys) / LDLR c.245G>A p.(Cys82Tyr)	Heterozygous / Heterozygous	LP/ P	Yes	No	Ankara
P64	16	NM_000384.3	APOB c.9201G>T p.(Lys3067Asn)	Heterozygous	VUS	Yes	Yes	Ankara
P65	17	NM_000527.5	LDLR c.1274del p.(Asn425ThrfsTer2)	Heterozygous	P	No	No	Ankara
P66	17	NM_000527.5	LDLR c.2403_2406del p.(Leu802AlafsTer126)	Heterozygous	P	Yes	No	Kastamonu
P67	18	NM_000527.5	LDLR c.2477_2493del p.(Pro826HisfsTer4)	Heterozygous	P	Yes	Yes	Ankara
P68	18	NM_000527.5	LDLR c.343C>T p.(Arg115Cys) / LDLR c.245G>A p.(Cys82Tyr)	Heterozygous / Heterozygous	P/ P	Yes	No	Ankara
P69	18	NM_000384.3	APOB c.6913A>G p.(Ile2305Val)	Heterozygous	VUS	Yes	No	Ankara
P70	20	NM_000237.3	LPL c.557G>A p.(Gly186Glu)	Homozygous	LP	Yes	Yes	Syria
P71	21	NM_000527.5	LDLR c.9199A>G p.(Lys3067Glu)	Heterozygous	VUS	Yes	Yes	Manisa
P72	22	NM_000527.5	LDLR c.1618G>A p.(Ala540Thr)	Heterozygous	P	No	Yes	Ankara
P73	25	NM_000527.5	LDLR c.504C>A p.(Asp168Glu)	Heterozygous	P	No	No	Ankara
P74	27	NM_000527.5	LDLR c.372del p.(Gln125SerfsTer81)	Heterozygous	LP	No	No	Ankara
P75	27	NM_000527.5	LDLR c.826T>C p.(Cys276Arg)	Heterozygous	LP	No	Yes	Syria
P76	27	NM_000384.3	APOB c.6734C>T p.(Thr2245Ile)	Heterozygous	VUS	No	No	Hatay
P77	27	NM_005912.3	MC4R c.990C>A p.(Ser330Arg)	Heterozygous	VUS	No	No	Ankara
P78	29	NM_001371904.1	APOA5 c.586G>T p.(Glu196Ter)	Homozygous	LP	Yes	No	Ankara
P79	29	NM_000527.5	LDLR c.1502C>T p.(Ala501Val)	Heterozygous	P	No	No	Cankiri
P80	29	NM_000527.5	LDLR c.1729T >C p.(Trp577Arg)	Heterozygous	P	Yes	No	Batman
P81	31	NM_000527.5	LDLR c.386A>T p.(Asp129Val)	Heterozygous	LP	Yes	Yes	Istanbul
P82	31	NM_000527.5	LDLR c.1048C>T p.(Arg350Ter)	Heterozygous	P	Yes	No	Ankara
P83	33	NM_001371904.1	APOA5 c.16_39del p.(Ala6_Ala13del)	Homozygous	P	Yes	Yes	Ankara
P84	34	NM_000527.5	LDLR c.1102T>G p.(Cys368Gly)	Homozygous	P	Yes	Yes	Afyon
P85	34	NM_000384.3	APOB c.8258C>G p.(Pro2753Arg)	Heterozygous	VUS	Yes	Yes	Erzurum

Patient ID	Age	Transcript (NM_)	Variant (HGVS)	Zygosity	ACMG Classification	SBS	DCLN Score	Geographic origin
P86	35	NM_000527.5	LDLR c.622G>A p.(Glu208Lys)	Heterozygous	P	Yes	No	Samsun
P87	35	NM_000527.5	LDLR c.761A>C p.(Gln254Pro)	Homozygous	P	Yes	Yes	Ankara
P88	35	NM_000237.3	LPL c.226T>A p.(Phe76Ile)	Heterozygous	VUS	Yes	No	Ankara
P89	38	NM_000527.5 / NM_000384.3	LDLR c.1247G>A p.(Arg416Gln) / APOB c.9175C>T p.(Arg3059Cys)	Heterozygous / Heterozygous	P/ VUS	Yes	Yes	Antalya
P90	39	NM_000527.5	LDLR Delesyon (10.42 Kbp)	Heterozygous	LP	No	Yes	Konya
P91	39	NM_000516.7	GNAS c.331_336del p.(Lys111_Lys112del)	Heterozygous	VUS	No	No	Ankara
P92	40	NM_000527.5	LDLR c.138C>G p.(Cys46Trp)	Heterozygous	LP	No	Yes	Denizli
P93	42	NM_000527.5 / NM_000384.3	LDLR c.2237-1G>T/ Heterozygous duplication encompassing APOB exons 1–28	Heterozygous	VUS	No	No	Ankara
P94	43	NM_000384.3	APOB c.10268T>C p.(Val3423Ala)	Heterozygous	VUS	No	No	Ankara
P95	44	NM_000527.5	LDLR c.939C>A p.(Cys313Ter)	Heterozygous	P	Yes	Yes	Ankara
P96	45	NM_000527.5	LDLR c.1678A>T p.(Ile560Phe)	Heterozygous	P	Yes	Yes	Mardin
P97	45	NM_000527.5	LDLR c.2312-3C>A	Heterozygous	P	Yes	Yes	Ankara
P98	45	NM_000384.3	APOB c.10256A>G p.(Lys3419Arg)	Heterozygous	VUS	No	No	Corum
P99	46	NM_000384.3	APOB c.9201G>T p.(Lys3067Asn)	Heterozygous	VUS	No	No	Kirikkale
P100	47	NM_000527.5	LDLR c.1729T >C p.(Trp577Arg)	Heterozygous	P	Yes	Yes	Ankara
P101	48	NM_000527.5	LDLR c.1729T >C p.(Trp577Arg)	Heterozygous	P	Yes	Yes	Kirikkale
P102	50	NM_000527.5	LDLR c.2477_2493del p.(Pro826HisfsTer4)	Heterozygous	P	No	No	Ankara
P103	51	NM_000527.5	LDLR c.1274del p.(Asn425ThrfsTer2)	Heterozygous	P	No	No	Tokat
P104	52	NM_001371904.1	APOA5 c.586G>T p.(Glu196Ter)	Heterozygous	P	Yes	Yes	Gumushane
P105	53	NM_000527.5	LDLR c.1048C>T p.(Arg350Ter)	Heterozygous	P	Yes	Yes	Ankara
P106	53	NM_000384.3	APOB c.2728G>C p.(Gly910Arg)	Heterozygous	VUS	No	No	Ankara
P107	54	NM_000527.5	LDLR c.664T>C p.(Cys222Arg)	Heterozygous	P	Yes	Yes	Artvin
P108	54	NM_000384.3	APOB c.400G>T p.(Ala134Ser)	Heterozygous	VUS	Yes	No	Corum
P109	55	NM_000527.5	LDLR c.2054C>T p.(Pro685Leu)	Heterozygous	P	No	No	Kirsehir
P110	56	NM_000384.3	APOB c.10315A>T p.(Asn3439Tyr)	Homozygous	VUS	Yes	Yes	Ankara
P111	58	NM_000527.5	LDLR c.1729T >C p.(Trp577Arg)	Heterozygous	P	No	No	Yozgat
P112	58	NM_000527.5	LDLR c.1729T>C p.(Trp577Arg)	Heterozygous	P	Yes	Yes	Ankara
P113	59	NM_000527.5	LDLR c.418_426del p.(Glu140_Ser142del)	Heterozygous	LP	No	No	Ankara

**Patient ID**	**Age**	Transcript (NM_)	**Variant (HGVS)**	**Zygosity**	**ACMG Classification**	**SBS**	**DCLN Score**	**Geographic origin**
P114	59	NM_000527.5	LDLR c.1061A>G p.(Asp354Gly)	Heterozygous	P	Yes	Yes	Karabuk
P115	61	NM_000527.5	LDLR c.1678A>T p.(Ile560Phe)	Heterozygous	P	No	No	Ankara
P116	61	NM_000527.5	LDLR c.1678A>T p.(Ile560Phe)	Heterozygous	P	Yes	Yes	Ankara
P117	62	NM_000237.3	LPL c.953A>G p.(Asn318Ser)	Heterozygous	VUS	No	No	Cankiri
P118	71	NM_000527.5	LDLR c.2312-3C>A	Heterozygous	P	Yes	Yes	Ankara
P119	72	NM_000527.5 / NM_174936.4	LDLR c.504C>A p.(Asp168Glu) / PCSK9 c.1399C>G p.(Pro467Ala)	Heterozygous / Heterozygous	P/ LP	Yes	Yes	Corum

DLCN Score: Dutch Lipid Clinic Network Score; SBS: Simon Broome Score; ACMG: American College of Medical Genetics; P: Pathogenic; LP: Likely Pathogenic; VUS: Variant of Unknown Significance

Supplementary Table S5. Detailed evaluation of novel variants. Case-based descriptions and variant-level evidence summaries for novel sequence variants detected in genes associated with lipid metabolism and familial dyslipidemias. Variant nomenclature follows HGVS recommendations.

**Variant narratives**
***APOA5* c.586G>T, p.(Glu196Ter) – heterozygous, proband A**
A stop-gain variant in *APOA5* (c.586G>T; p.Glu196Ter) was identified in a 52-year-old female. The proband had markedly elevated triglyceride (TG) levels, and her daughter had a history of hyperlipidemia. The variant is located in exon 3 (the final exon) of the 3-exon *APOA5* gene and is predicted to remove more than 10% of the encoded transcript/protein sequence. However, the gene-level loss-of-function (LoF) intolerance metrics (low pLI together with a relatively high observed/expected ratio for LoF) are consistent with potential tolerance to haploinsufficiency. The variant was not observed in gnomAD and was therefore considered novel. Most available in silico prediction tools suggested a non-damaging/benign impact, although computational prediction is inherently limited for truncating variants, particularly those in terminal exons that may escape nonsense-mediated decay (NMD). *APOA5* expression is predominantly hepatic. No previously reported pathogenic variant with the identical amino-acid change was found in the literature.
***LDLR* c.2477_2493del, p.(Pro826HisfsTer4)**
A novel 17-bp deletion in *LDLR* (c.2477_2493del; p.Pro826HisfsTer4) was detected in an 18-year-old female with elevated total cholesterol. Both parents had a history of hyperlipidemia. Subfraction data did not reveal a pronounced isolated increase beyond the elevated total cholesterol. The variant lies in exon 17 of the 18-exon *LDLR* gene and is not within the last 50 nucleotides of the exon, supporting a predicted premature termination sufficiently upstream of the final exon-exon junction to trigger NMD and result in LoF. Although population-based LoF constraint metrics for *LDLR* (low pLI and a relatively high LoF observed/expected ratio) can appear permissive, ClinGen has curated *LDLR* as haploinsufficient, and the overwhelming majority of reported *LDLR* LoF variants are classified as pathogenic. The variant is absent from gnomAD. While variant-specific in silico predictions were not available for this deletion, reports of pathogenic/likely pathogenic variants affecting nearby residues within the same region have been described, supporting functional relevance of the affected segment.
***LDLR* c.372del, p.(Gln125SerfsTer81)**
A novel single-nucleotide deletion in *LDLR* (c.372del; p.Gln125SerfsTer81) was identified in a 27-year-old female with a maternal history of hyperlipidemia; lipid profile data for the proband were not available. The variant is located in exon 4 and introduces a frameshift with a premature termination codon, consistent with LoF. Multiple pathogenic or likely pathogenic *LDLR* variants have been reported in the same regional context, suggesting functional sensitivity of this portion of the gene. As with other *LDLR* truncating variants, the ClinGen haploinsufficiency curation supports clinical relevance of LoF alleles. The variant is not present in gnomAD, and variant-specific *in silico* prediction results were not available.
***LDLR* c.1496C>T, p.(Ser499Phe)**
The *LDLR* missense variant c.1496C>T (p.Ser499Phe) was found in a 9-year-old girl with a family history of early-onset cardiac disease. Both total cholesterol and LDL-cholesterol were markedly elevated. The variant is located in exon 10 within an annotated functional region of *LDLR*. Across multiple in silico predictors, the amino-acid substitution was consistently predicted to be deleterious. Although *LDLR* shows limited population-level missense constraint (reported missense Z-score ~0.12), missense variation in *LDLR* is frequently pathogenic in clinical practice. The variant was not observed in gnomAD. Importantly, pathogenic variants affecting the same codon but resulting in different amino-acid substitutions have been reported, supporting functional importance of this residue.

LDLR c.140A>G, p.(Asp47Gly) - homozygous

A novel *LDLR* missense variant (c.140A>G; p.Asp47Gly) was detected in an 8-year-old girl presenting with elevated total cholesterol with LDL-cholesterol predominance; family history information was unavailable. The variant was observed in the homozygous state and maps to exon 2. In silico prediction tools unanimously supported a deleterious effect on protein function. The allele was not present in gnomAD. Prior literature reports have described pathogenic and/or conflicting (VUS) interpretations for other variants affecting the same residue, suggesting that this position may be functionally critical.

LDLR c.386A>T, p.(Asp129Val)

The *LDLR* missense variant c.386A>T (p.Asp129Val) was identified in a 31-year-old female with hypercholesterolemia characterized by LDL-cholesterol predominance. The proband's mother and sibling were reported to have hyperlipidemia. This variant is located in exon 4. Multiple in silico predictors uniformly suggested a deleterious impact. The variant was not observed in gnomAD. Additionally, pathogenic or conflicting (VUS) variants have been reported at the same amino-acid residue, further supporting functional relevance of this site.

LDLR c.418_426del, p.(Glu140_Ser142del)

An in-frame deletion in *LDLR* (c.418_426del; p.Glu140_Ser142del) was found in a 59-year-old female with elevated total cholesterol, predominantly driven by LDL-cholesterol; a family history of hyperlipidemia was reported. The variant is located in exon 4 and removes three amino acids without altering the reading frame. The allele was not present in gnomAD and was considered novel. Reports of pathogenic or likely pathogenic variants affecting residues in the same region have been described, suggesting functional constraint across this segment. Consistent with this, missense and small in-del variants in LDLR are frequently classified as pathogenic, supporting potential clinical relevance of this in-frame event.

CFH c.2237-1G>T

A novel splice-acceptor variant in *CFH* (c.2237-1G>T) was detected in a 42-year-old male with elevated total cholesterol and LDL-cholesterol predominance; family history was unavailable. The variant affects the canonical acceptor site of exon 15 in the 22-exon *CFH* gene and was absent from gnomAD. In silico splicing prediction tools indicated a strong splice-altering effect (SpliceAI score 0.94, predicted splice-altering, strong). Gene-level constraint metrics support LoF intolerance for *CFH* (pLI=1; LoF observed/expected approximately 0.33), and LoF *CFH* variants have been reported as pathogenic in established *CFH*-related disease contexts. Whether this specific variant triggers NMD cannot be determined with certainty based on sequence context alone; the predicted transcript consequence corresponds to a truncation affecting less than 10% of the protein-coding sequence (~4.8% transcript loss). In addition, the individual had a suspected heterozygous duplication spanning *APOB* exons 1-28. While there is sufficient evidence for *APOB* haploinsufficiency, evidence for clinically meaningful triplosensitivity remains limited. On the basis of the available evidence, the *CFH* variant was classified as a variant of uncertain significance (VUS).

PCSK9 c.145G>A, p.(Glu49Lys)

The *PCSK9* missense variant c.145G>A (p.Glu49Lys) was identified in a 15-year-old male with elevated total cholesterol and LDL-cholesterol predominance. The variant is located in exon 1 of the 12-exon *PCSK9* gene and was absent from gnomAD. *PCSK9* shows limited population-level constraint against missense variation (missense Z-score ~0.27), and the fraction of pathogenic missense variants reported for *PCSK9* is relatively low compared with benign variation. Consistent with this, available in silico predictors did not support a deleterious impact on gene/protein function, and there were no prior reports of a pathogenic variant affecting the same residue.

APOB c.10256A>G, p.(Lys3419Arg)

A novel *APOB* missense variant (c.10256A>G; p.Lys3419Arg) was detected in a 45-year-old male with elevated total cholesterol and LDL-cholesterol predominance and a family history of cardiac disease. The variant is located in exon 26 of the 29-exon *APOB* gene and was not observed in gnomAD. *APOB* shows low population-level missense constraint (reported missense Z-score approximately -1.7), and the proportion of pathogenic missense variants in *APOB* is relatively low compared with benign missense variation. Accordingly, in silico predictors did not indicate a deleterious effect, and no published pathogenic variants affecting the same residue were identified.

***MC4R* c.990C>A, p.(Ser330Arg)**

The *MC4R* missense variant c.990C>A (p.Ser330Arg) was identified in a 27-year-old female with dyslipidemia characterized primarily by marked hypertriglyceridemia, together with elevated total cholesterol; family history was present but incompletely characterized. *MC4R* is a single-exon gene with low missense constraint (missense Z-score ~-0.36). Despite this, *MC4R* missense variants are frequently reported as pathogenic in the literature in the context of *MC4R*-related phenotypes. For the present variant, in silico predictors did not support a deleterious impact, and no prior pathogenic reports affecting the same amino-acid residue were found. The variant was absent from gnomAD.

***APOA5* c.586G>T, p.(Glu196Ter) - homozygous (Proband B)**

The same *APOA5* stop-gain variant (c.586G>T; p.Glu196Ter) was also detected in a second proband, a 29-year-old female, in the homozygous state. The lipid phenotype consisted of elevated total cholesterol with prominent hypertriglyceridemia. Family history was unclear. As noted above, the variant is located in the terminal exon of *APOA5*, is absent from gnomAD, and lacks a previously reported pathogenic counterpart with the identical amino-acid change. The observation of homozygosity in the context of pronounced hypertriglyceridemia may be compatible with a dose-dependent or recessive contribution, although the gene-level LoF tolerance metrics suggest that additional genetic or environmental modifiers may influence expressivity.

***LPL* c.226T>A, p.(Phe76Ile)**

A novel missense variant in LPL (c.226T>A; p.Phe76Ile) was identified in a 35-year-old male with dyslipidemia dominated by markedly elevated triglycerides, alongside increased total cholesterol. The variant is located in exon 2 of the 10-exon LPL gene and was absent from gnomAD. In silico predictors suggested a potentially deleterious effect. While *LPL* shows limited population-level missense constraint (missense Z-score approximately -0.5), missense variants in *LPL* are frequently reported as pathogenic in the literature. No previously reported pathogenic variant affecting the same residue was identified.

***LDLR* c.1231_1242del, p.(Lys411_Leu414del)**

An in-frame deletion in *LDLR* (c.1231_1242del; p.Lys411_Leu414del) was detected in an 8-year-old girl with elevated total cholesterol and prominent LDL-cholesterol elevation; family history was reported but not detailed. The variant lies in exon 9 and removes four amino acids without shifting the reading frame. The allele was not present in gnomAD. Prior reports of pathogenic *LDLR* variants involving neighboring residues support functional sensitivity of this region. Given that missense and in-frame in-del variants in *LDLR* are frequently classified as pathogenic, this novel in-frame deletion may be clinically relevant, pending segregation and/or functional evidence.

Supplementary Discussion (brief)

Across the set of novel findings, variants in *LDLR* accounted for the largest proportion, aligning with the established centrality of *LDLR* dysfunction in familial hypercholesterolemia phenotypes. The two novel frameshift variants (*LDLR* c.372del and *LDLR* c.2477_2493del) are predicted to result in loss of function and, in light of ClinGen haploinsufficiency curation and the predominance of pathogenic classifications among reported *LDLR* LoF alleles, represent the strongest candidates for clinical relevance among the variants described. Several additional *LDLR* variants (missense substitutions at p.Asp47, p.Asp129 and p.Ser499, and in-frame deletions affecting p.Glu140_Ser142 and p.Lys411_Leu414) cluster within

extracellular regions where previously reported pathogenic or conflicting variants have been described, supporting functional constraint at these residues/segments; nevertheless, segregation and/or functional assays would materially strengthen interpretation for the novel alleles. In contrast, the novel missense variants identified in PCSK9 and APOB show limited computational support for deleterious impact and occur in genes with low missense constraint metrics, emphasizing the importance of avoiding over-interpretation of rare missense changes in the absence of corroborating functional or segregation evidence. The recurrent *APOA5* stop-gain variant (p.Glu196Ter), observed in both heterozygous and homozygous states in individuals with prominent hypertriglyceridemia, is notable because it lies in the terminal exon and *APOA5* exhibits gene-level metrics compatible with relative tolerance to haploinsufficiency; the observed phenotypes may therefore reflect zygosity, modifier alleles, and/or environmental contributions, and warrant follow-up studies. Finally, the *CFH* splice-acceptor variant had strong in silico evidence for splicing disruption in a LoF-constrained gene, but its relevance to the proband's lipid phenotype remains uncertain, highlighting the need for phenotype matching and for considering additional genomic findings (e.g., the suspected *APOB* duplication) when classifying variants of uncertain significance.

Interpretation statement: The summaries above reflect the clinical and computational information provided and do not replace gene/disease-specific ACMG/AMP variant interpretation with full access to segregation, functional studies, and complete phenotyping.
